# Comorbid illnesses are associated with altered adaptive immune responses to SARS-CoV-2

**DOI:** 10.1172/jci.insight.146242

**Published:** 2021-03-22

**Authors:** Krystle K.Q. Yu, Stephanie Fischinger, Malisa T. Smith, Caroline Atyeo, Deniz Cizmeci, Caitlin R. Wolf, Erik D. Layton, Jennifer K. Logue, Melissa S. Aguilar, Kiel Shuey, Carolin Loos, Jingyou Yu, Nicholas Franko, Robert Y. Choi, Anna Wald, Dan H. Barouch, David M. Koelle, Douglas Lauffenburger, Helen Y. Chu, Galit Alter, Chetan Seshadri

**Affiliations:** 1 Department of Medicine, University of Washington School of Medicine, Seattle, Washington, USA.; 2 Ragon Institute of MGH, MIT and Harvard, Cambridge, Massachusetts, USA.; 3 PhD program in Immunology and Virology, University of Duisburg-Essen, Essen, Germany.; 4 PhD program in Virology, Division of Medical Sciences, Harvard University, Boston, Massachusetts, USA.; 5 Department of Biological Engineering, Massachusetts Institute of Technology, Cambridge, Massachusetts, USA.; 6 Center for Virology and Vaccine Research, Beth Israel Deaconess Medical Center, Harvard Medical School, Boston, Massachusetts, USA.; 7 Providence Medical Group, Everett, Washington, USA.; 8 Department of Epidemiology and; 9 Department of Laboratory Medicine and Pathology, University of Washington School of Medicine, Seattle, Washington, USA.; 10 Vaccine and Infectious Diseases Division, Fred Hutchinson Cancer Research Center, Seattle, Washington, USA.; 11 Department of Global Health, University of Washington, Seattle, Washington, USA.; 12 Benaroya Research Institute, Seattle, Washington, USA.

**Keywords:** COVID-19, Immunology, Adaptive immunity, Beta cells, T cells

## Abstract

Comorbid medical illnesses, such as obesity and diabetes, are associated with more severe COVID-19, hospitalization, and death. However, the role of the immune system in mediating these clinical outcomes has not been determined. We used multiparameter flow cytometry and systems serology to comprehensively profile the functions of T cells and antibodies targeting spike, nucleocapsid, and envelope proteins in a convalescent cohort of COVID-19 subjects who were either hospitalized (*n* = 20) or not hospitalized (*n* = 40). To avoid confounding, subjects were matched by age, sex, ethnicity, and date of symptom onset. Surprisingly, we found that the magnitude and functional breadth of virus-specific CD4^+^ T cell and antibody responses were consistently higher among hospitalized subjects, particularly those with medical comorbidities. However, an integrated analysis identified more coordination between polyfunctional CD4^+^ T cells and antibodies targeting the S1 domain of spike among subjects who were not hospitalized. These data reveal a functionally diverse and coordinated response between T cells and antibodies targeting SARS-CoV-2, which is reduced in the presence of comorbid illnesses that are known risk factors for severe COVID-19.

## Graphical abstract

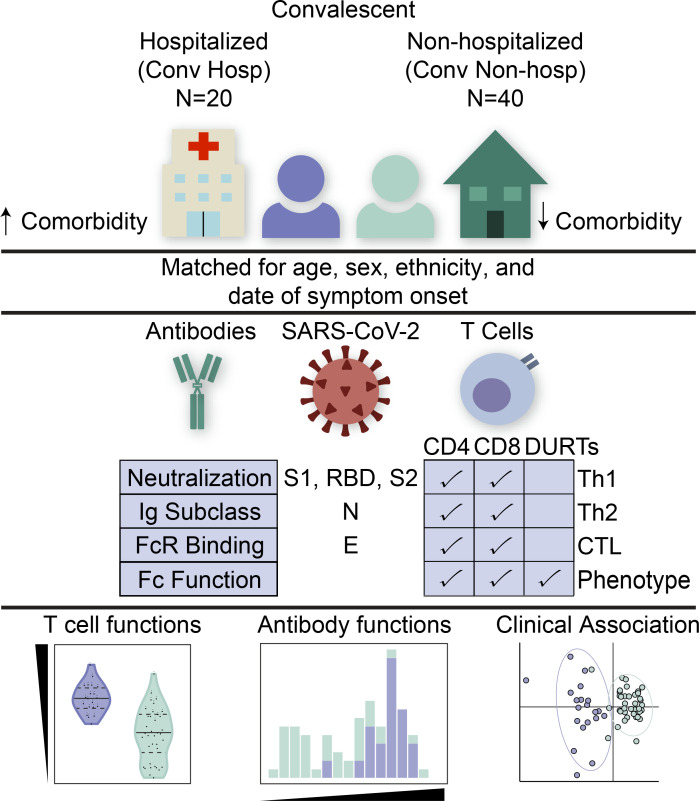

## Introduction

Severe Acute Respiratory Syndrome Coronavirus 2 (SARS-CoV-2) causes Coronavirus Disease 2019 (COVID-19), which is responsible for over 2 million deaths since its discovery in 2019 (1, 2). The clinical course of COVID-19 is variable and ranges from asymptomatic or mild disease to acute respiratory distress syndrome (ARDS) and death (3). Epidemiologic studies have revealed several factors, such as advanced age, male sex, and nonwhite ethnicity (4–6), that are associated with adverse clinical outcomes, including hospitalization. The presence of medical comorbidities, such as obesity, diabetes, and heart disease, are also associated with more severe disease (7–9). Viral load at diagnosis is an independent predictor of mortality, and duration of viral shedding was longer among hospitalized patients who died (10, 11).

Several studies have identified lymphopenia and an increase in proinflammatory cytokines associated with hospitalization for COVID-19 (12–15). Neutralizing antibody titers have also been associated with increased disease severity (16–18). Detailed studies using flow and mass cytometry, as well as single cell RNA sequencing, have revealed perturbations in several subpopulations of T cells and B cells among patients with severe COVID-19 (19–23). However, T cells and antibodies execute a range of functions only after encountering their cognate antigens, so the pursuit of further details regarding their role in the pathogenesis of COVID-19 has required looking beyond bulk populations of lymphocytes. Several studies have investigated whether virus-specific T cell and antibody responses are associated with disease severity (24–26). However, these studies have not comprehensively examined the functions of antigen-specific T cells and have not been designed to robustly examine associations with clinical risk factors. A major limitation has been confounding due to demographic factors, such as age and sex, as well as the date of symptom onset, all of which can influence associations with immune status independent of COVID-19 (27, 28). For example, some studies reported differences between acutely ill patients, healthy controls, and recovered donors, but the healthy controls were significantly younger and recovered donors had blood drawn much later in their illness course (19, 25).

In this study, we sought to overcome these limitations in study design and to more comprehensively examine the functional profiles of antigen-specific immune responses and their association with risk factors and clinical outcomes after COVID-19. We leveraged a large cohort of convalescent donors, including individuals recruited as candidate donors for convalescent plasma donation (29) in Seattle, Washington, USA, where SARS-CoV-2 community transmission was first described in the United States (30). We selected study participants that were either hospitalized (*n* = 20) or not hospitalized (*n* = 40) after matching for age, sex, ethnicity, and date of symptom onset. Archived serum was used to compare neutralizing antibody titers, as well as Ig levels, Fc receptor (FcR) binding, and Fc effector functions targeting full spike (S), S1, S2, receptor binding domain (RBD), and nucleocapsid (N) proteins. Archived peripheral blood mononuclear cells (PBMCs) were used to compare frequencies and phenotypes of conventional αβ T cells, as well as donor-unrestricted T cells (DURTs) (31). Finally, we compared the functional profiles of antigen-specific T cells targeting S1, S2, N, and envelope (E) proteins using intracellular cytokine staining (ICS). In nearly all the parameters tested, we consistently observed both higher magnitudes and increased functional breadth among hospitalized subjects, particularly those with medical comorbidities. However, T cell and antibody responses showed less correlation among hospitalized subjects. Our analysis reveals a qualitative shift in the adaptive immune response to SARS-CoV-2, which may be directly related to the presence of comorbid illnesses that are known risk factors for severe disease.

## Results

### Cellular and humoral dynamics in a matched cohort of convalescent COVID-19 subjects.

We utilized a cohort of convalescent COVID-19 subjects stratified by hospitalization status and matched for confounders most relevant for immune profiling studies — namely age, sex, and race/ethnicity (Table 1). We further matched for the interval between the self-reported date of symptom onset and specimen collection, as this could also influence kinetics of SARS-CoV-2–specific immune responses (32). This resulted in a final set of COVID-19 subjects who were either hospitalized (*n* = 20) or not hospitalized (*n* = 40) and from whom plasma and PBMCs were collected at a median of approximately 50 days after symptom onset (Table 1). Quantitative viral load information was available from 16 subjects and varied over a wide range (Supplemental Table 1; supplemental material available online with this article; https://doi.org/10.1172/jci.insight.146242DS1). Consistent with prior reports, comorbid diseases were more frequently observed among hospitalized subjects (*P* = 0.001, Fisher’s exact test) (7–9).

**Figure 1:**
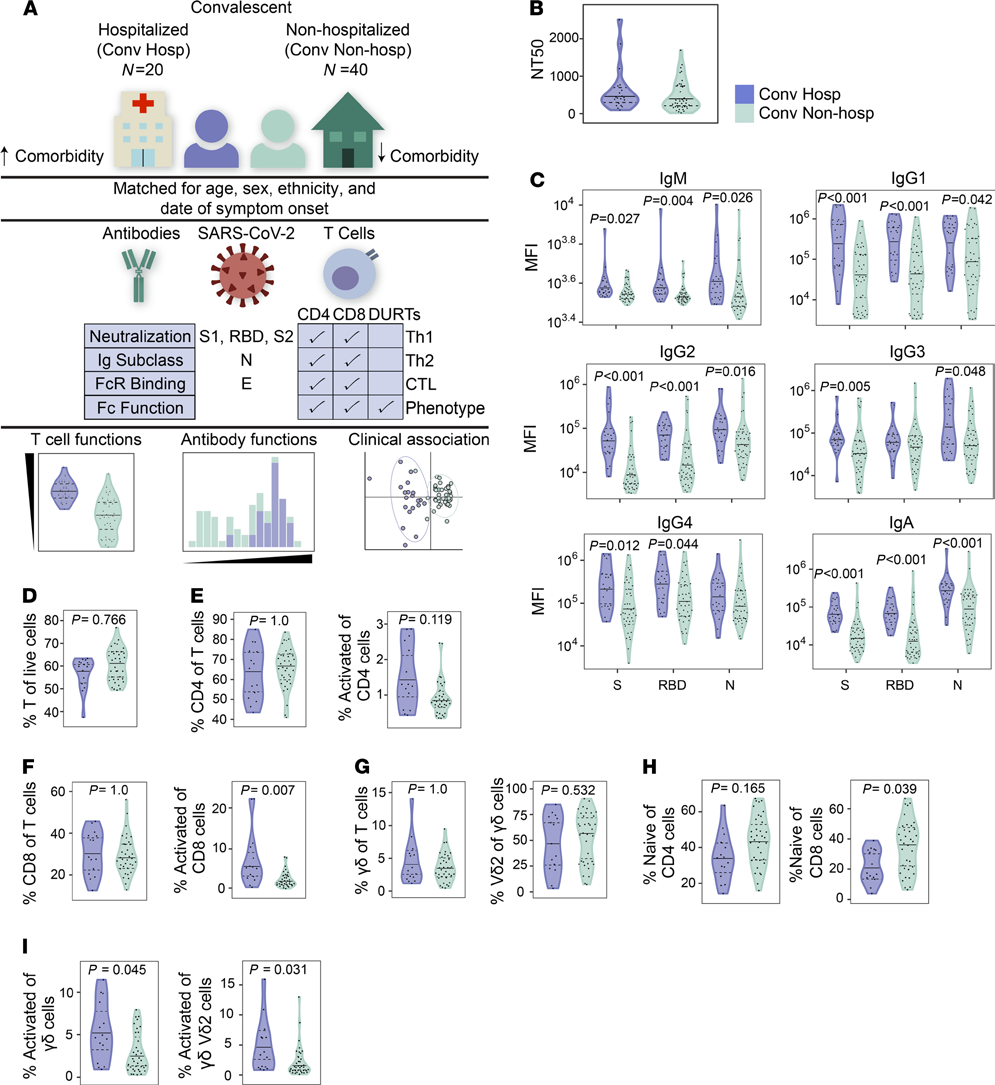
Cellular and humoral dynamics in a matched cohort of convalescent COVID-19 subjects. (**A**) Study schema. Archived peripheral blood mononuclear cells (PBMC) and plasma from COVID-19 study subjects who were previously hospitalized (purple, *n* = 20) or nonhospitalized (green, *n* = 40) were selected based on matching for age, sex, ethnicity, and date of symptom onset. Samples were comprehensively profiled for SARS-CoV-2–specific T cell and antibody phenotypes and functions. Data were analyzed to identify differences between the groups and to build a classifier. DURTs, donor-unrestricted T cells. (**B**) Antibody neutralization titers were compared between hospitalized and nonhospitalized subjects. NT50 denotes the concentration of serum required to achieve 50% of the maximum neutralization in the assay. (**C**) Comparison of antibody subclass and isotype levels against spike (S), receptor binding domain (RBD), and nucleocapsid (N) antigens between groups. (**D**) Flow cytometric analysis comparing the percent of total CD3^+^ T cells between groups. (**E** and **F**) Among CD3^+^ T cells, the percent of CD4^+^ T cells (**E**), CD8^+^ T cells (**F**), and activation statuses as defined by coexpression of HLA-DR and CD38 was compared between groups. (**G**) The frequency of γδ T cells as a percent of total CD3^+^ T cells and Vδ2 T cell frequencies as a percent of γδ T cells are compared between hospitalized and nonhospitalized patients. (**H**) The percentage of naive CD4^+^ and CD8^+^ T cells as defined by coexpression of CD45RA and CCR7 is assessed between groups. (**I**) The frequencies of activated γδ and Vδ2 T cells are compared between groups. NT50, Ig titers, and T cell frequencies were compared between groups using Mann-Whitney *U* tests, followed by correction for multiple hypothesis testing using the Bonferroni method. Median, 25th, and 75th quartiles are indicated for violin plots. If not shown, *P* values for Mann-Whitney *U* tests were not significant.

We used multiparameter flow cytometry and system serology to comprehensively study the functional profiles of T cells and antibodies targeting SARS-CoV-2 S, N, and E proteins (Figure 1A). We also examined the neutralization activity of patient sera and noted that these were not associated with hospitalization status (Figure 1B). This result suggested that other humoral or T cell functional profiles may be associated with clinical outcomes in COVID-19 subjects. We examined the magnitude of Ig subclasses targeting the S, the S1, S2 or RBD of spike, and N, which were broadly stable in both groups of subjects over time (Supplemental Figure 1, A and B). IgG1, IgG2, IgG4, and IgA titers against S, S1, S2, and RBD were significantly higher among hospitalized subjects (Figure 1C and Supplemental Figure 2A). Moreover, all Ig subclasses except IgG4-targeting N were also significantly higher among hospitalized subjects, and we have previously demonstrated that anti-N antibodies are a marker of disease severity (Figure 1C) (33). These results show that antibody subclass titers, rather than neutralization, may be associated with clinical outcomes after COVID-19.

### Antibody functional profiles are associated with hospitalization after COVID-19.

**Figure 2:**
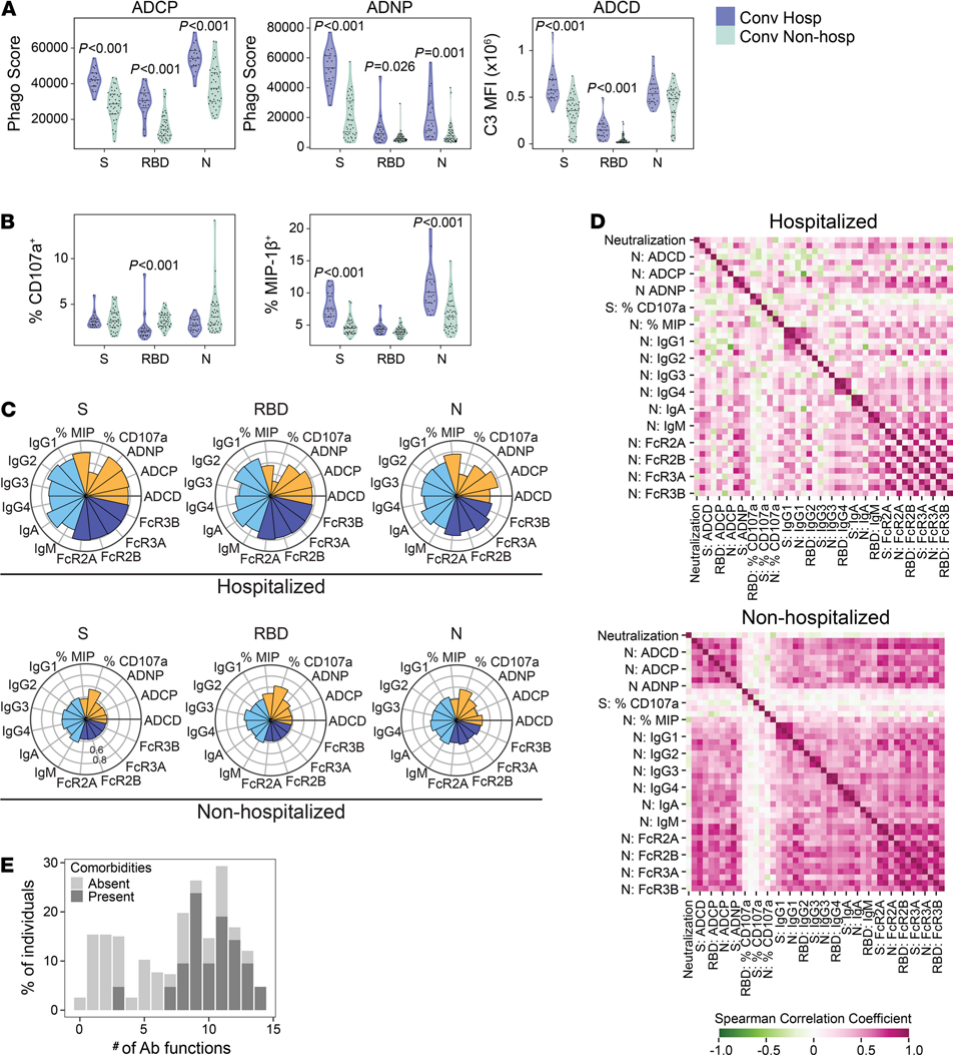
Antibody functional profiles are associated with hospitalization after COVID-19. SARS-CoV-2–specific antibody phenotypes and functional profiles were compared between hospitalized (purple, *n* = 20) and nonhospitalized (green, *n* = 40) COVID-19 study subjects. (**A** and **B**) Antibody-dependent cellular phagocytosis (ADCP), antibody-dependent neutrophil phagocytosis (ADNP), antibody-dependent complement deposition (ADNP) (**A**), and NK cell activation as measured by MIP-1β secretion or CD107a expression (**B**) against spike (S), receptor binding domain (RBD), and nucleocapsid (N) was quantified and compared between groups. (**C**) Nightingale rose graphs show the distribution around the mean profiles of antibody features for S, RBD, and N among hospitalized and nonhospitalized subjects. Each flower petal represents a SARS-CoV-2–specific antibody measurement. The size of the petal depicts the percentile above/below the mean across both groups. The colors indicate type of feature: antibody function (orange), titer (light blue), and Fc-receptor binding (dark blue). (**D**) The correlation matrix shows the Spearman correlation coefficient for antibody features separately in hospitalized and nonhospitalized subjects. Pink indicates a positive correlation, whereas green indicates a negative correlation. (**E**) Polyfunctional antibody profiles were compared between subjects with and without comorbidities. To determine polyfunctionality, an individual’s response was noted to be functional if it was above the median response for the cohort. Per person, the number of positive functions was summed, resulting in a polyfunctionality score per individual. Polyfunctional scores are displayed as percent positivity of the whole cohort. Antibody phenotypes and effector functions excluding neutralization were compared across groups using Mann-Whitney *U* tests, followed by correction for multiple hypothesis testing using the Bonferroni method. Median, 25th, and 75th quartiles are indicated for violin plots. If not shown, *P* values for Mann-Whitney *U* tests were not significant.

To follow up these differences in Ig subclass, we examined several Fc-binding specificities and Fc-dependent effector functions. Fc-receptors (FcRs) specificities FcR2A, FcR2B, FcR3A, and FcR3B binding S, S1, S2, RBD, and N were significantly higher among hospitalized subjects (Supplemental Figure 2B). Antibody-dependent cellular phagocytosis (ADCP), antibody-dependent neutrophil phagocytosis (ADNP), and antibody-dependent complement deposition (ADCP) against S, RBD, and N was significantly increased among hospitalized subjects (Figure 2A). Notably, while MIP-1β secretion by NK cells was increased among hospitalized subjects, NK cell degranulation measured by CD107a expression was elevated among nonhospitalized subjects (Figure 2B). To obtain a qualitative summary of the differences in antigen-specific humoral responses between groups, we visualized Ig subclass, Fc-binding specificity, and Fc-effector functions targeting S, RBD, and N using nightingale rose graphs (Figure 2C). The results show consistently higher levels of measured analytes among hospitalized subjects, with the exception of CD107a expression on NK cells. We next examined the correlation of antibody profiles independently in hospitalized and nonhospitalized subjects. The correlation with neutralization titers in both groups was low, supporting our analysis of nonredundant aspects of the SARS-CoV-2–specific antibody response. This was surprising, given previous findings that IgG antibodies to RBD are correlated with neutralization titers (34, 35). Relative to nonhospitalized subjects, hospitalized subjects demonstrated lower correlation among antibody titers, Fc-specificities, and Fc-effector functions (Figure 2D). This difference was robust to subsampling in order to account for the unequal sample sizes in each group (Supplemental Figure 3). Finally, we calculated a polyfunctionality score for each individual for S, RBD, and N over the 6 antibody functionality readouts against 3 SARS-CoV-2 antigens. Subjects with comorbidities were able to activate a robust polyfunctional antibody response against S, RBD, and N in comparison with subjects without comorbidities (Figure 2E). Taken together, these results reveal qualitative and quantitative increases in several aspects of the SARS-CoV-2–specific antibody response among hospitalized subjects with comorbidities, many of which are likely the result of differences in innate immune system activation and T cell help.

### Activated CD8^+^ and γδ T cells are associated with hospitalization after COVID-19.

To investigate the role of T cells, we used multiparameter flow cytometry to quantify the frequencies and phenotypes of conventional and DURT populations, such as invariant NK T (iNKT) cells, mucosal-associated invariant T (MAIT) cells, and γδ T cells (31). In our matched cross-sectional analysis, we noted that the frequency of CD3^+^, CD4^+^, and CD8^+^ T cells did not vary significantly over time since symptom onset or between hospitalized and nonhospitalized subjects (Figure 1, D–F, and Supplemental Figures 1C). We also found no difference in the frequency of γδ T cells, iNKT cells, or MAIT cells or in B cells, monocytes, or NK cells (Figure 1G and Supplemental Figure 5, A and B). However, the frequency of activated CD8^+^ T cells was significantly higher among hospitalized subjects, which is consistent with prior reports (Figure 1F) (19, 36, 37). The frequency of naive CD8^+^ T cells was also lower among hospitalized subjects, suggesting increased differentiation to an effector phenotype in severe COVID-19; however, no difference in the frequency of naive CD4^+^ T cells was observed between groups (Figure 1H). Among total γδ T cells, the frequency of activated γδ T cells was higher among hospitalized subjects independent of expression of the Vδ2 gene segment (Figure 1I). The frequency of activated CD4^+^, CD8^+^, and γδ T cells was broadly steady over time since symptom onset, which is in contrast to some reports (Supplemental Figure 1D and Supplemental Figure 5C) (36). These data confirm and extend published studies by revealing the durability of differences in activated CD8^+^ and γδ T cell but not CD4^+^ T cell populations in a matched cross-sectional analysis stratified by hospitalization status.

### IFN-γ–independent CD4^+^ T cell responses to SARS-CoV-2 structural antigens.

**Figure 3:**
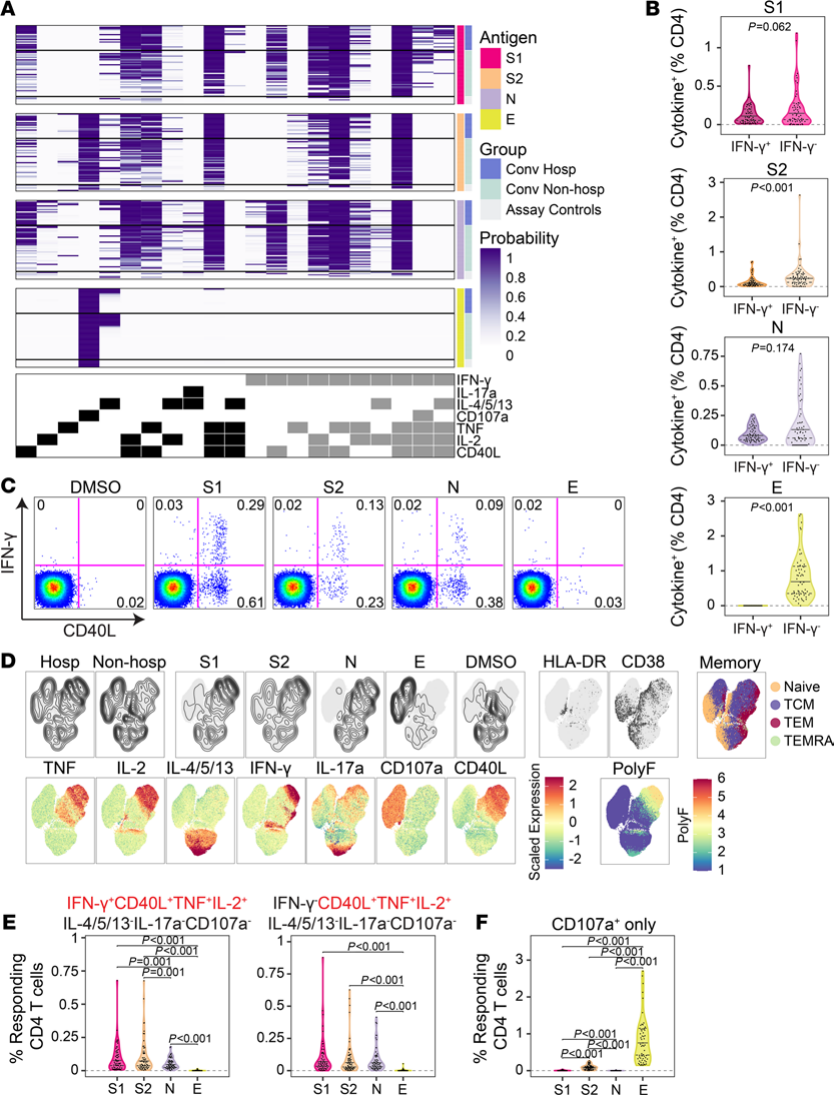
IFN-γ–independent CD4 T cell responses to SARS-CoV-2 structural antigens. (**A**) Intracellular cytokine staining was used to profile the functions of CD4^+^ T cells specific for the S1 and S2 domains of spike, nucleocapsid (N), and envelope small membrane protein (E). Data were analyzed using COMPASS, and results are displayed as a probability heatmap in which the rows represent study subjects and the columns represent CD4^+^ T cell functional subsets. The depth of shading within the heatmap represents the probability of detecting a response above background. In the column legend, white indicates absence and black/gray indicates presence of a function. (**B**) Background subtracted magnitudes of CD4^+^ T cell responses stratified by the presence of IFN-γ. (**C**) Representative bivariate flow cytometry plots showing the expression of IFN-γ and CD40L following stimulation. (**D**) Cells expressing any of the functional profiles identified by COMPASS were aggregated across all subjects prior to performing dimensionality reduction with UMAP. Plots are stratified and colored according to hospitalization status, stimulation, effector function, memory markers (naive, CD45RA^+^CCR7^+^; central memory [TCM], CD45RA^–^CCR7^+^; effector memory [TEM], CD45RA^–^CCR7^–^; and effector memory RA [TEMRA], CD45RA^+^CCR7^–^), and activation markers (HLA-DR, CD38). Polyfunctionality (PolyF) was calculated as the number of cytokines gated positive for each cell. (**E**) Magnitudes of CD4^+^ T cells expressing a CD40L^+^IL-2^+^TNF^+^ functional profile in the presence or absence of IFN-γ are compared across stimulations. (**F**) Magnitudes of CD4^+^ T cells expressing CD107a in the absence of all other functions are compared across stimulations. Wilcoxon signed-rank tests were used to compare frequencies between groups in **B** and **E**. **E** reports Bonferroni-corrected *P* values, but **B** is unadjusted. Median, 25th, and 75th quartiles are indicated for violin plots. If not shown, *P* values were not significant. *n* = 60 in all panels.

We next investigated the functional profiles of SARS-CoV-2–specific T cells. PBMCs were stimulated with overlapping peptide pools targeting the S1 or S2 domain of S, N, or E. We used ICS to identify antigen-specific T cells expressing IL-2, IL-4/5/13, IL-17a, IFN-γ, TNF, CD107a, and CD40L (Supplemental Figure 4). To ensure the detection of polyfunctional T cell subsets that may be present at low frequencies, we employed Combinatorial Polyfunctionality Analysis of Antigen-Specific T Cell Subsets (COMPASS) (38). Among 128 possible functional profiles, we detected 21 antigen-specific CD4^+^ T cell subsets across all 4 peptide pool stimulations (Figure 3A). Notably, the probability of detecting a particular response varied according to the antigen. For example, several profiles containing 3 or 4 functions were readily detected after stimulation with S1, S2, or N but not E. However, the 2 profiles containing 5 functions (IFN-γ, IL-14/5/13, TNF, IL-2, and CD40L) were only detected after stimulation with S1. Stimulation with E resulted in a CD107a monofunctional profile that was also observed after stimulation with S2 (Figure 3A).

Because COMPASS only reports the probability of detecting a particular response, we next examined the magnitude of T cell responses stratified by the presence of IFN-γ. Notably, 11 (52%) of the 21 CD4^+^ T cell functional profiles identified by COMPASS did not contain IFN-γ (Figure 3A). We found nearly equivalent numbers of IFN-γ^+^ and IFN-γ^–^ T cells after stimulation with S1 or N. However, more T cells expressed IFN-γ–independent functions after stimulation with S2 and E (Figure 3, B and C). These data suggest that a substantial fraction of the SARS-CoV-2–specific T cell response could be missed by conventional assays, such as IFN-γ ELISPOT (39). We used uniform manifold approximation and projection (UMAP) to examine qualitative associations between hospitalization status, stimulation, and T cell functional profiles. Hospitalization appeared to be associated with responses to S1, S2, and N, though there was overlap with nonhospitalized subjects (Figure 3D). The degree of polyfunctionality appeared to be associated with hospitalization, which was also suggested by COMPASS (Figure 3, A and D). Among the 21 functional profiles identified by COMPASS, CD4^+^ T cells simultaneously expressing CD40L, IL-2, and TNF were detected at the greatest magnitudes, regardless of the presence of IFN-γ, and were highest after stimulation with S1 or S2 (Figure 3E and Supplemental Figure 6). By contrast, approximately 1% of CD4^+^ T cells expressed CD107a independently of IFN-γ after stimulation with E (Figure 3F). Finally, CD4^+^ T cells with a detectable cytokine response predominantly expressed a CCR7^+^CD45RA^–^ central memory phenotype, but very few demonstrated coexpression of the activation markers HLA-DR and CD38 (Figure 3D). These data demonstrate the functional diversity of CD4^+^ T cell responses to SARS-CoV-2 structural antigens driven in large part by IFN-γ–independent profiles that are not typically the focus of vaccine immunogenicity or epitope mapping studies (40–42).

### Functional diversity of CD4^+^ T cell responses to SARS-CoV-2 are associated with hospitalization.

**Figure 4:**
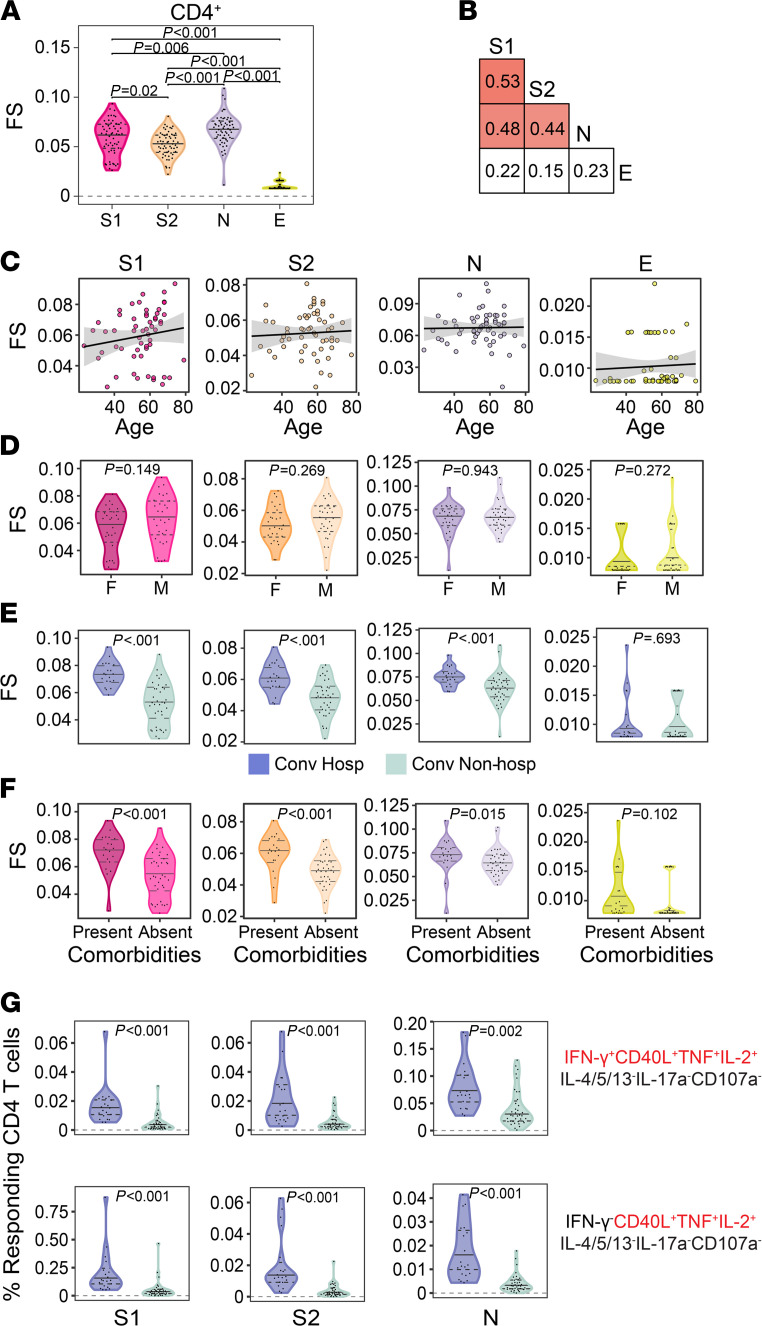
Functional diversity of CD4^+^ T cell responses to SARS-CoV-2 are associated with hospitalization. (**A**) The CD4^+^ T cell functionality score (FS) was determined by COMPASS and compared across all 4 stimulation conditions. (**B**) Two-way correlations of FS were calculated between stimulations. Colored squares indicate a statistically significant correlation (*P* < 0.05). (**C**–**E**) For each stimulation, we examined the association with age (**C**), sex (**D**), and hospitalization status (**E**). The black lines on the scatter plots represent best fit linear regression lines, and the gray-shaded areas represent the 95% CI of the predicted means. (**F**) CD4 functionality scores for each stimulation were compared in the presence and absence of comorbidities. (**G**) Background corrected magnitudes of CD4^+^ T cells expressing a CD40L^+^IL-2^+^TNF^+^ functional profile in the presence or absence of IFN-γ are compared between groups after stimulation with S1, S2, and N. CD4 functionality scores were compared using Wilcoxon signed-rank tests or Mann-Whitney *U* tests, followed by correction for multiple hypothesis testing using the Bonferroni method except for **D** and **F**. Supplemental Figure 6 shows all the functional profiles that were compared with obtain *P* values reported in **G**. Median, 25th, and 75th quartiles are indicated for violin plots. If not shown, *P* values were not significantly different. *n* = 60 in all panels.

Since UMAP revealed a qualitative association between T cell functional profile and hospitalization, we wanted to next explore that relationship quantitatively. To accomplish this, we used COMPASS to calculate a functionality score (FS), which summarizes the functional breadth for each subject and stimulation into a continuous variable that can be incorporated into standard statistical models (38). Among CD4^+^ T cells, we found the highest FS after stimulation with N, followed by S1, S2, and then E (Figure 4A). However, the correlation between stimulations was modest, even between S1 and S2, confirming the importance of examining each antigen and functional domain independently (Figure 4B). CD4 FS were not associated with age or sex for any of the antigens tested (Figure 4, C and D). Notably, the functional breadth of CD4^+^ T cell responses was stable over time (Supplemental Figure 1E). Finally, we investigated whether FS were associated with clinical risk factors and outcomes. We found higher FS to S1, S2, and N but not E among hospitalized subjects and in the presence of medical comorbidities (Figure 4, E and F). We examined this association using magnitudes of polyfunctional (CD40L^+^IL-2^+^TNF^+^) CD4^+^ T cells and found the same to be true independent of the production of IFN-γ (Figure 4G). Thus, our data reveal that increased functional breadth of CD4^+^ T cell responses to S and N are associated with known risk factors for severe COVID-19 independent of the production of IFN-γ.

### CD8^+^ T cell responses to SARS-CoV-2 structural antigens are not associated with hospitalization.

**Figure 5:**
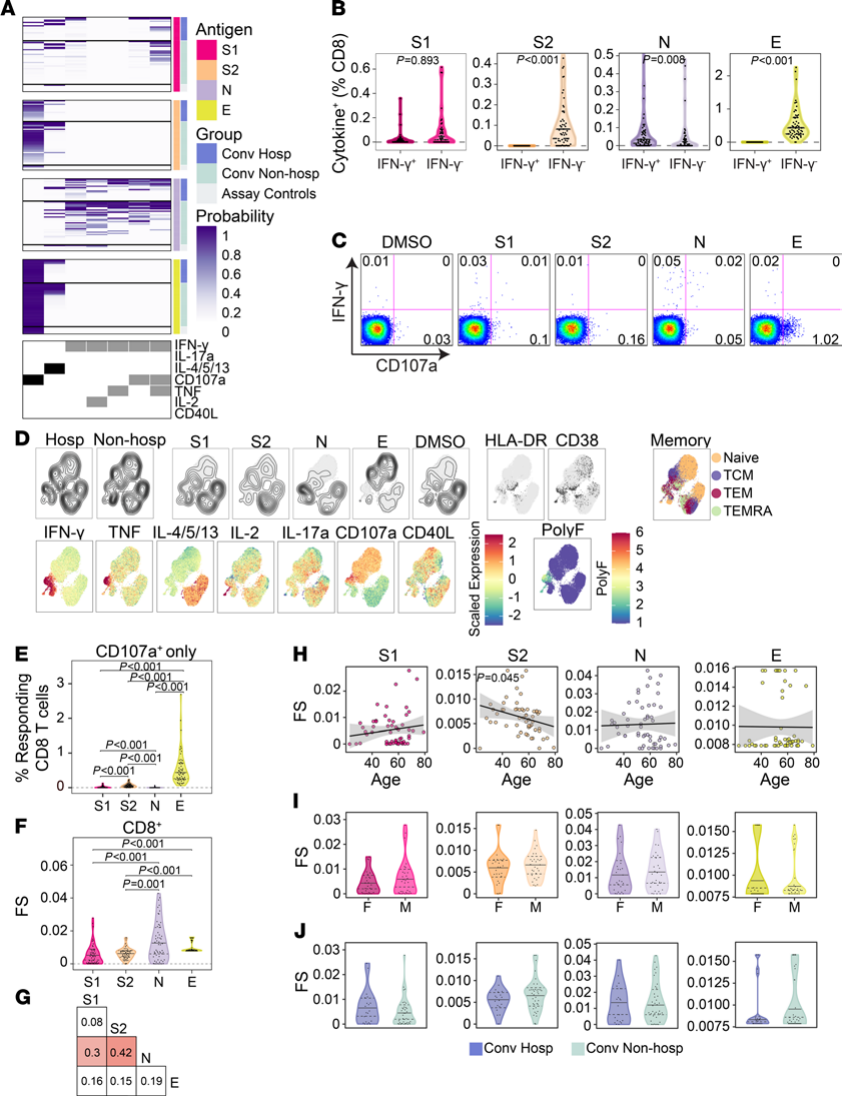
CD8^+^ T cell responses to SARS-CoV-2 structural antigens are not associated with hospitalization. (**A**) Intracellular cytokine staining was used to profile the functions of CD8^+^ T cells specific for the S1 and S2 domains of spike, nucleocapsid (N), and envelope small membrane protein (E). Results of COMPASS are displayed as a heatmap in which rows represent study subjects and columns represent CD8^+^ T cell functional subsets. The depth of shading within the heatmap represents the probability of detecting a response above background. In the column legend, white indicates absence and black/gray indicates presence of a function. (**B**) Background subtracted magnitudes of CD8^+^ T cell responses stratified by the presence of IFN-γ. A single outlier is not displayed for S2 and N. (**C**) Representative bivariate flow cytometry plots showing the expression of IFN-γ and CD107a following stimulation. (**D**) Cells expressing any of the functional profiles identified by COMPASS were aggregated across all subjects prior to UMAP. (**E**) Magnitudes of CD8^+^ T cells expressing CD107a in the absence of other functions. (**F**) The CD8^+^ T cell functionality score (FS) as determined by COMPASS. (**G**) Two-way correlations of FS were calculated between stimulations. Colored squares indicated a statistically significant correlation (*P* < 0.05). (**H**–**J**) For each stimulation, we examined the association with age (**H**), sex (**I**), and hospitalization status (**J**). Black lines on the scatter plots represent best fit linear regression lines, and the gray-shaded areas represent the 95% CI of the predicted means. Data were analyzed using Wilcoxon signed-rank tests (**B**, **E**, and **F**) and Mann-Whitney *U* tests (**I** and **J**) and were corrected for multiple hypothesis testing using the Bonferroni method. Median, 25th, and 75th quartiles are indicated for violin plots. If not shown, *P* values were not significant. *n* = 60 in all panels.

We next explored the functional breadth of CD8^+^ T cell responses and its association with hospitalization. In contrast to the CD4^+^ T cell response, COMPASS analysis identified 7 T cell subsets, of which only 2 lacked IFN-γ (Figure 5A). IFN-γ–independent T cell responses were dominant after stimulation with S2 and E (Figure 5, A and B) and were characterized by expression of CD107a (Figure 5, A and C). Both UMAP and COMPASS revealed polyfunctional profiles consisting of IFN-γ, IL-2, and TNF that were largely detected after stimulation with N in both hospitalized and nonhospitalized subjects (Figure 5, A and D). Similar to CD4^+^ T cells, CD107a monofunctional CD8^+^ T cells were mostly detected after stimulation with S2 and E (Figure 5E). Cytokine-producing CD8^+^ T cells were distributed across effector memory, central memory, and effector memory RA (TEMRA) phenotypes and did not coexpress activation markers HLA-DR and CD38 (Figure 5D). Analysis of CD8 FS revealed the greatest breadth after stimulation with N and very little correlation between antigens (Figure 5, F and G). Again, we noted a surprisingly poor correlation between S1 and S2 that was driven by the dominance of polyfunctional responses to S1 and CD107a monofunctional responses to S2 (Figure 5, A and G). Only S2 FS were negatively correlated with age (Figure 5H). Finally, none of the stimulations were associated with sex, days after symptom onset, or hospitalization (Figure 5, I and J, and Supplemental Figure 1F). Together, these data reveal that a thorough assessment of CD8 functional responses requires assays that examine more than IFN-γ and that IFN-γ production and cytotoxic function are poorly correlated, even between the S1 and S2 domains of spike glycoprotein.

### Antigen-specific T cell and antibody responses are less coordinated among hospitalized subjects.

**Figure 6:**
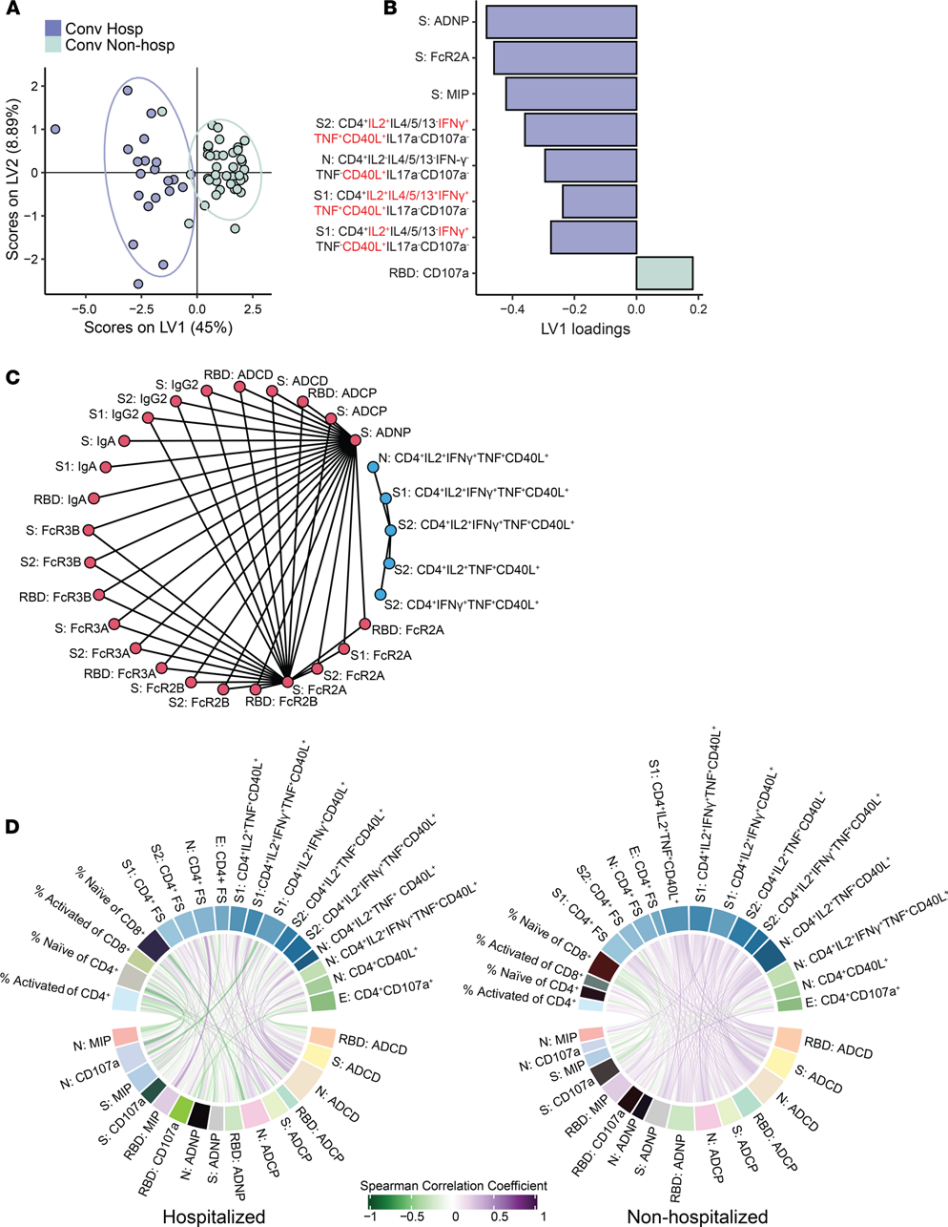
A classifier based on antibody and T cell features predicts hospitalization status. (**A**) Partial least squares discriminant analysis (PLS-DA) was used to identify features that could discriminate between hospitalized (purple) and nonhospitalized (green) subjects. The PLS-DA scores plot shows the separation between groups using the first 2 latent variables (LVs). Each dot represents an individual, and ellipses correspond to the 95% data ellipse for each group. (**B**) The bar plot shows the LV1 loadings of the LASSO-selected features for the PLS-DA ranked based on their variable importance in projection (VIP) score. Features are color coded according to the group in which they are enriched — i.e., the group with the higher average values of the feature. (**C**) The correlation network was generated from all the features correlated with LASSO-selected features. A cutoff with Spearman *P* > 0.8 and *P* < 0.005 is shown. A cutoff of Spearman *P* > 0.8 with a Benjamini-Hochberg adjusted *P* <0.05 was set, and only connections outside of this cutoff are shown. The graph was generated using R package network (72, 74). (**D**) The chord diagram generated using the R package circlize (73) shows Spearman correlations between T cell features and antibody-dependent effector functions for nonhospitalized and hospitalized subjects. Spearman correlations are shown as links that carry the color of the average correlation coefficient between the functional antibody features and T cell measurements. The arc length of each segment is automatically scaled to the number of correlating segments it pairs with. To exclude potential bias caused by the number of subjects in nonhospitalized (*n* = 40) and hospitalized (*n* = 20) groups, per-group Spearman correlations were calculated by sampling 10 subjects 100 times and computing the average of the Spearman correlation coefficients for each antibody feature–T cell measurement pair.

Our results indicate consistently higher magnitudes and increased functional breadth of several antibody and T cell features among hospitalized subjects. Thus, we next sought to identify the minimum set of features that could differentiate between hospitalized and nonhospitalized subjects. We used least absolute shrinkage and selection operator (LASSO) and identified 8 features that consistently distinguished the 2 clinical groups via partial least squares discriminant analysis (PLS-DA) (Figure 6A and Supplemental Figure 7). With the exception of the induction of CD107a expression on NK cells by anti-RBD antibodies, all features were consistently enriched among hospitalized subjects (Figure 6B). When we examined the correlation between the selected features and all measured features, we noted that ADNP and FcR2A targeting spikes were highly correlated with other features of humoral immunity (Figure 6C). Furthermore, the 5 CD4 polyfunctional T cell features did not correlate with each other or with the humoral features, indicating a nonredundant contribution of T cell functions to the classification. Finally, we examined how T cell and antibody features correlated with each other in the 2 groups. Among nonhospitalized subjects, we noted more significant positive correlations (*n* = 196 of 255) between T cell and antibody features as compared with subjects who were hospitalized (*n* = 136 of 255), even when the 2 groups were downsampled to account for the different sample sizes (Figure 6D and Supplemental Figure 3). These data reveal increased coordination of antigen-specific T cell and antibody responses to SARS-CoV-2 among nonhospitalized subjects, despite reduced magnitudes and functional breadth, compared with subjects who were hospitalized.

## Discussion

In summary, we performed a cross-sectional study comprehensively examining the functional profiles of T cells and antibodies targeting SARS-CoV-2 S, N, and E proteins in convalescent subjects who were either hospitalized or not hospitalized. We consistently found the magnitude and functional breadth of measured responses to be higher among hospitalized subjects and in the presence of medical comorbidities. However, these responses were less correlated with each other when compared with nonhospitalized subjects. These data support the possibility that medical comorbidities predispose to greater but a less coordinated and ultimately less effective response to SARS-CoV-2 infection.

In contrast to most studies in which T cells or antibodies are studied in isolation, we comprehensively profiled and analyzed them together in the context of detailed clinical information. In almost every respect, we found that they track together and show high levels of coordination among nonhospitalized subjects. The lack of coordination observed among hospitalized subjects may reflect a failure to control the virus at early stages, resulting in increased inflammation and virus load. Comorbid diseases were overrepresented among hospitalized subjects, suggesting that they may be related to the increased functional breadth among T cells and antibodies that we describe here. Supporting this hypothesis are studies examining the effect of diabetes on adaptive immunity to *Mycobacterium* *tuberculosis* (43). These studies have shown increased production of antigen-specific Th1 and Th17 cytokines in the presence of chronic hyperglycemia, which is associated with an increased inflammatory state (44, 45). Whether SARS-CoV-2–specific T cell and antibody responses with increased functional breadth are the cause of poor clinical outcomes is not addressed by the cross-sectional design of our study and more definitively assessed in longitudinal studies or animal models. Another limitation of our study design is that samples were collected during convalescence, even among hospitalized subjects, which precludes our ability to define correlates of disease risk. This could be overcome with human challenge studies in which a detailed analysis of T cell and antibody responses could be prospectively correlated with clinical outcomes (46).

SARS-CoV vaccine studies in mouse models have revealed the detrimental effect of imbalanced Th1 and Th2 immunity (47, 48). By contrast, mRNA-1273 and BNT162b2 have been shown to be highly effective against SARS-CoV-2 and induce minimal Th2 immunity (49, 50). Our data reveal important nuances in this paradigm, including the preferential expansion of highly polyfunctional T cells targeting S1 in hospitalized subjects who express IL-4/5/13, TNF, CD40L, and IL-2 in the presence or absence of IFN-γ (Figure 3A and Supplemental Figure 6A). Notably, T cells with this profile targeting HIV envelope were identified as a correlate of protection in the RV144 vaccine trial (38). Whether the responses we observed here are induced by vaccination and associated with protection from COVID-19 remains to be determined.

Notably, we did not observe an association between neutralizing antibody titers and hospitalization in our study, which is consistent with one study but contrasts with other studies examining patients much earlier in their disease course (24, 25, 35, 51, 52). However, we did find that several functional attributes of spike-specific antibodies, including Ig subclass titers, were poorly correlated with neutralization yet associated with hospitalization. We have also previously shown that the ratio of S/N antibodies is more predictive of death among hospitalized subjects than neutralization titers (33). These data add to a growing body of literature showing that several attributes of virus-specific antibodies are associated with clinical outcomes, including hospitalization (53). In general, we found that Ig subclass titers, Fc specificity, and Fc-effector functions were lower among nonhospitalized subjects but were more highly correlated with each other compared with hospitalized subjects. These findings may be the result of differences in innate immune activation, which may contribute to increased viral clearance and lower antigen loads. Innate immunity is known to be impaired in older subjects and in the presence of comorbidities like diabetes (54).

Our data have several implications for the rollout of preventive vaccines for COVID-19. Phase I studies of subunit vaccines have quantified S-specific antibodies or neutralizing titers, as well as IFN-γ production by S-specific T cells as evidence of immunogenicity (41, 42). However, we show that neutralizing antibody titers are poorly correlated with several important functional qualities of S-specific antibodies. We also show that a significant fraction of the CD4^+^ T cell response to S does not include IFN-γ and depends on which domain is being examined. For example, CD4^+^ and CD8^+^ T cell responses to S2 were notable for having a cytotoxic phenotype compared with S1. In the integrated analysis, 8 T cell and antibody features primarily focused on S1 were sufficient to classify hospitalized subjects with near perfect accuracy. One interpretation of these data is that severe disease is associated with a prolonged stimulus to immunity. Phase I studies that report safety are typically tested on young, healthy volunteers who are not representative of the target populations for candidate COVID vaccine; these populations are likely older and with medical comorbidities (55). This is a particularly important concern, since several of the platforms being used, such as mRNA and adenoviral vectors, have limited experience in large clinical efficacy studies. An expanded analysis of the functions of vaccine-specific T cells and antibodies beyond what is required for regulatory approval will be required to understand the full benefits or risks of each approach.

## Methods

Further information can be found in Supplemental Methods.

### Study population

Whole blood samples were collected from individuals with laboratory-confirmed SARS-CoV-2 infection as part of a prospective longitudinal cohort study or as part of a protocol in support of expanded access to convalescent plasma for treatment of COVID-19 (ClinicalTrials.gov, NCT04338360). People 18 years or older with laboratory-confirmed SARS-CoV-2 infection were eligible for inclusion. From the prospective study, individuals included in this report were from 2 groups: previously hospitalized inpatients and nonhospitalized outpatients. Inpatients were hospitalized at Harborview Medical Center, University of Washington Medical Center or at Northwest Hospital (Seattle, Washington, USA) and were identified through a laboratory alert system. Patients who were recruited as inpatients were enrolled during their hospital admission and had samples collected during their hospitalization, regardless of the date of symptom onset. After hospital discharge, these participants were asked to present to an outpatient clinical research site approximately 30 days after symptom onset for follow-up. In-person follow-up only occurred if participants were asymptomatic as per Center for Disease Control and Prevention (CDC) guidelines. Outpatients were identified through a laboratory alert system, email and flyer advertising, and through positive COVID-19 cases reported by the Seattle Flu Study (30). Outpatients completed their enrollment, data collection questionnaire, and first blood draw at an outpatient clinic visit approximately 30 days after symptom onset (or positive test for asymptomatic individuals). All participants subsequently were asked to return at day 60 and then at day 90 or 120 for follow-up. From protocol NCT04338360, only subjects with a history of hospitalization were considered for inclusion in this report. Sociodemographic and clinical data were collected from chart review and from participants at the time of enrollment (56), including information on the nature and duration of symptoms, medical comorbidities, and care-seeking behavior (Supplemental Table 1). Separately, assay control samples were derived from a 2017 adult specimen repository study or obtained from Bloodworks Inc. To quantify the severity of COVID-19 disease, participants were classified on a Clinical Scale Assessment (CSA) scale ranging from 1 to 8, in which 1 is most severe and 8 is least severe (Supplemental Table 1).

### Functional antibody measurements

Bead-based assays were used to quantify ADCP, ADNP, and ADCD, as previously described (57–59). Fluorescent neutravidin beads (red for ADCD, yellow for ADNP and ADCP) (Thermo Fisher Scientific) were coupled to biotinylated SARS-CoV-2 antigens RBD, S, and N and incubated with diluted plasma (ADCP and ADNP, 1:100 dilution; ADCD, 1:10 dilution) for 2 hours at 37°C. For measuring monocyte phagocytosis, 2.5 × 10^4^ THP-1 cells (ATCC) were added per well and incubated for 16 hours at 37°C. For ADNP, ammonium-chloride-potassium (ACK) lysis was performed on whole blood from healthy blood donors (MGH blood donor center), and 5 × 10^4^ cells were added per well and incubated for 1 hour at 37°C. Then, a Pacific Blue (PacBlue) anti-CD66b detection antibody (clone G10F5 [RUO]; BioLegend) was used to stain neutrophils. To assess ADCP, lyophilized guinea pig complement (Cedarlane) was reconstituted and added to each well for 20 minutes at 37°C. Subsequently, a FITC-conjugated goat IgG fraction to guinea pig complement C3 (MP Biomedicals) was added to detect C3 binding. Following fixation, sample acquisition was performed via flow cytometry (Intellicyt, iQue Screener plus) utilizing a robot arm (PAA), and analysis occurred using Forecyt software. A phagocytosis score was calculated for ADCP and ADNP as (percentage of bead^+^ cells) × (MFI of bead^+^ cells) divided by 10,000. ADCD was reported as MFI of FITC C3 deposition.

For the measurement of antibody-dependent NK cell–activating functions, an ELISA-based surrogate assay was employed as described previously (60). Briefly, plates were coated with 3 μg/mL of antigen (S, RBD, and N), and samples were added at a 1:50 dilution and incubated for 2 hours at 37°C. NK cells were isolated the day prior via RosetteSep (Stemcell Technologies) from healthy buffy coats (MGH blood donor center) and rested overnight in 1 ng/mL IL-15 (Stemcell Technologies). A total of 5 × 10^4^ NK cells was then added to the ELISA plates containing the immune complexes and incubated for 5 hours at 37°C in the presence of CD107a PE-Cy5 (clone H4A3, BD Biosciences), GolgiStop (BD Biosciences), and BFA (Sigma-Aldrich). Following the incubation, cells were fixed with Perm A (Invitrogen) and stained for surface markers with anti–CD16 APC-Cy7 (clone 3G8), anti–CD56 PE-Cy7 (clone B159), and anti–CD3 PacBlue (clone SP34-2) antibodies (BD Biosciences). Subsequently, cells were permeabilized using Perm B (Thermo Fisher Scientific), and ICS with anti–IFN-γ FITC (clone 4S.B3) and anti–MIP-1β PE (clone D21-1351) (BD Biosciences) was performed. NK cells were defined as CD3^–^, CD16^+^, and CD56^+^. Data were reported as percentage of cells positive for CD107a, MIP-1β, or IFN-γ. All functional assays were performed in duplicate with 2 donors if applicable.

### Flow cytometry of T cells

PBMC samples were thawed in warm thaw media consisting of RPMI 1640 (Thermo Fisher Scientific) supplemented with 10% FBS (Hyclone; R10), and 2 μL/mL Benzonase (MilliporeSigma) sterile-filtered and centrifuged at 250*g* for 10 minutes at room temperature. The supernatant was decanted, and the viable cells were enumerated using the Guava easyCyte (MilliporeSigma) with guavaSoft 2.6 software. The cells were centrifuged at 250*g* for 10 minutes at room temperature and rested overnight at a density of 2 million cells/mL. The following day, the cells were enumerated using the Guava easyCyte and analyzed using 2 multiparameter flow cytometry assays: Surface marker staining (described in Supplemental Methods) and ICS.

For ICS, we stimulated cells with overlapping peptide pools (15 mers overlapping by 11 amino acids) targeting the S1 or S2 domains of spike glycoprotein, N, or E proteins (JPT Peptide Technologies). The S1 pool spans the N-terminal amino acid residues (1–643 amino acids, 158 peptides) of spike glycoprotein, while the S2 pool spans the C-terminal amino acid residues (633–1273 amino acids, 157 peptides). Each peptide pool was reconstituted with 40 or 50 μL of pure DMSO (Sigma-Aldrich) and then diluted with PBS for a final concentration of 100 μg/mL in 16% DMSO/84% PBS or 20% DMSO/80% PBS. PBMC were plated at a density of up to 1 × 10^6^ cells/well in a 96-well U-bottom plate and stimulated with 1 μg/mL of each peptide in the pool, or 0.25 μg/mL Staphylococcal Enterotoxin Type B (SEB) (List Biological Laboratories Inc.) or 0.2% DMSO (Sigma-Aldrich). In addition to antigen, the stimulation cocktail consisted of 1 μg/mL anti-CD28/49d (BD Biosciences), 10 μg/mL Brefeldin A (BFA; Sigma-Aldrich), GolgiStop (BD Biosciences) prepared according to manufacturer’s instructions, and anti–CD107a PE-Cy7 (clone H4A3, BD Biosciences). The cells were stimulated for 6 hours at 37°C, after which EDTA (Sigma-Aldrich) was added at a final concentration of 2 mM. Samples were then stored at 4°C overnight. The following day, PBMCs were washed twice with PBS then stained for 20 minutes at room temperature with Fixable Aqua viability dye (Invitrogen) prepared according to manufacturer’s instructions. A preparation of anti–CCR7 BV711 antibody (clone 150503, BD Biosciences) in FACS buffer was centrifuged at 10,000*g* for 5 minutes and then added to the cells for 30 minutes at 37°C. At the end of the incubation period, PBMCs were washed twice with FACS buffer and then incubated for 10 minutes at room temperature with 1× FACS Lyse (BD Biosciences). After lysis, the cells were washed with FACS buffer twice and then permeabilized by incubating for 10 minutes at room temperature with 1× FACS Perm II (BD Biosciences). The PBMC were again washed twice with FACS buffer and then stained with the remaining markers for 30 minutes at 4°C before being washed with FACS buffer: anti–CD3 ECD (clone UCHT1, Beckman Coulter); anti–CD4 APC-H7 (clone L200), anti–CD8β BB700 (clone 2ST8.5H7), anti–CD38 BV605 (clone HB7), anti–HLA-DR BUV395 (clone G46-6), anti–CD40L/CD154 PE-Cy5 (clone TRAP1), anti–CD45RA BUV737 (clone HI100), anti–IFN-γ BV421 (clone B27), anti–TNF FITC (clone MAb11), anti–IL-2 PE (clone MQ1-17H12), and anti–IL-4 APC (clone MP4-25D2) (BD Biosciences); and anti–CD14 BV785 (clone M5E2), anti–CD19 BV785 (clone SJ25C1), anti–IL-5 APC (clone TRFK5), anti–IL-13 APC (clone JES10-5A2), and anti–IL-17a Alexa Fluor 700 (clone BL168) (BioLegend). Finally, samples were fixed with 1% paraformaldehyde (Electron Microscopy Solution) and washed with PBS. They were then resuspended in PBS supplemented with EDTA at a final concentration of 2 mM and stored at 4°C until acquisition. For all flow cytometry experiments, study groups were evenly distributed in each batch, and operators were not blinded to study group assignments.

### Statistics

#### Flow cytometry data analysis.

Initial compensation, gating, and quality assessment of flow cytometry data were performed using FlowJo version 9.9.6 (FlowJo, TreeStar Inc.) for T cell data or Forecyt software (Intellicyt) for the antibody data. Representative gating trees for the surface marker and ICS panels are shown in Supplemental Figure 4. The surface marker and ICS flow cytometry data were then processed using the OpenCyto framework in the R programming environment (61). Samples with poor viability defined on the basis of low CD3 counts (<10,000 cells) or low CD4 counts (<3,000 cells) were excluded from analysis. For the ICS panel, data from 20 convalescent hospitalized and 37 convalescent nonhospitalized subjects were ultimately analyzed. For the surface marker panel, data from 15 convalescent hospitalized and 36 convalescent nonhospitalized subjects were analyzed.

To achieve a comprehensive and unbiased analysis of the functional profiles of antigen-specific T cells, we used COMPASS (38). COMPASS uses a Bayesian hierarchical framework to model all observed cell subsets and select those most likely to have antigen-specific responses. COMPASS naturally accounts for high background so that a cell subset with high background will have lower response probabilities compared with a similar subset with low background. Notably, COMPASS reports only the probability of detecting a particular T cell functional profile, rather than the absolute magnitude, which we calculated separately. For a given subject, COMPASS was also used to compute a FS that summarizes the entire functionality profile into a single continuous variable that can be used for standard statistical modeling (e.g., regression). For the data presented here, COMPASS was applied to each of the antigen stimulations separately for CD4^+^ and CD8^+^ T cells. Each one of the analyses was unbiased and considered all of the 128 possible boolean combinations of cytokine functions. Subjects with a high probability of response across many subsets were accordingly assigned a high FS. Magnitudes of T cell responses were calculated independent of COMPASS as the proportion of gated events in the stimulated condition minus the proportion of gated events in the unstimulated condition. Statistics were performed using background subtracted magnitudes, although data are plotted as the maximum of zero or this value. The R package ComplexHeatmap (62) was used to visualize COMPASS posterior probabilities of response. R packages corrplot and ggpubr, among others, were also use for analysis (63, 64).

UMAP was performed on all CD4^+^ or CD8^+^ events that were preselected from COMPASS-identified boolean subsets using the uwot package in R (65, 66), with the following parameters: spread = 9, min_dist = 0.02. The following markers were used in the UMAP analysis: CD3, CD4, CD8b, TNF, CD107a, CD40L/CD154, IL-2, IL-17a, IL-4/5/13, IFN-γ, CD45RA, CCR7, CD38, and HLA-DR. Fluorescence intensities of each marker were scaled within each batch to achieve a mean of zero and SD of 1 prior to UMAP.

The flow cytometry data supporting this publication are available at ImmPort (https://www.immport.org) under study accession SDY1680. The code to complete flow cytometry data analyses, including COMPASS, can be found at https://github.com/seshadrilab/Correlates_Severe_COVID19_ICS (branch name, master; Commit ID, a524f59f88f34df19306c43396598a02860ddf92) and https://github.com/seshadrilab/Correlates_Severe_COVID19_Surface_Markers (branch name, master; Commit ID, 1f421a8c8c8f2cd919c669febdbecc6fafd9c29a).

#### Integrated analysis of T cell and antibody functional profiles.

Classification models were trained to discriminate subjects between hospitalized and nonhospitalized subjects using all the measured humoral and T cell responses. Models were built with an approach similar to what we have previously published, using a combination of the LASSO for feature selection and then classification using PLS-DA with the LASSO-selected features (33, 60). The set of model inputs comprised functional and biophysical humoral responses and T cell responses to the SARS-CoV-2 antigens RBD, S, and N. In order to focus the analysis on biologically interesting and nonredundant parameters, we chose to include T cell parameters if they were significantly enriched in the hospitalized group — since none were enriched in the nonhospitalized group — followed by further downselection in the case of the ICS data for subsets expressing 3 or more cytokines or high magnitude of response. We also included the ICS CD4 Envelope CD107a subset and the CD4 Envelope FS score due to its surprisingly strong signal in our panel. Only functional antibody measurements were included in the coordination analysis. Input data were scaled and centered. Missing values on T cell responses were imputed using k-nearest neighbors, using the R package “DMwR” (version 0.4.1, knnImputation function) (67). Model robustness was assessed using 5-fold cross-validation. For each cross-validation run, subjects were randomly stratified into 5 subsets, ensuring that both groups were represented in each subset, with 4 subsets serving as the training set and the fifth as the test set. Each subset served as the test set once; therefore, each individual was in the test fold exactly once for each cross-validation run. For each test fold, LASSO-based feature selection was performed on logistic regression using the 4 subsets designated as the training set for that fold. Fold-specific LASSO was repeated 10 times, and features, which are selected 9 times out of 10, were identified as selected features. Using these selected features, a fold-specific PLS-DA was trained on training data for that fold. A set of predicted group labels were recorded for each subset. The first 2 latent variables (LVs) from a PLS-DA model trained on the LASSO-selected features were visualized. LVs are compound variables composed of the LASSO-selected features. For visualization, 95% data ellipses were calculated. Features were ordered according to their variable importance in projection (VIP) score, a score that is higher for features that contribute more to the model. Analyses were performed using R version 4.0.2 (2020-06-22).

Significance of model performance was evaluated using “negative control” models of permuted data and randomly selected size-matched features. The repetitions of 5-fold cross-validation generated a distribution of model classification accuracies. Corresponding model accuracy distributions were measured for 2 negative control models. The first approach consisted of permutation testing by randomly shuffling the group labels, within the cross-validation framework described above (i.e., a cross-validation framework matched to the actual model) (68). The second approach was to randomly select a set of features the same size as the LASSO-selected feature set. These control processes were repeated 100 times to generate a distribution of model accuracies observed in the context of permuted data and randomly selected, size-matched feature sets. The predicted group label for each subject was compared with the true group label to obtain a classification accuracy. Exact *P* values were obtained as the tail probability of the true classification accuracy in the distribution of control model classification accuracies. Because one of the LASSO-selected features (ADNP Spike) was highly correlated with 54% of all features, we further assessed the performance of randomly selected features by selecting only from the remaining 46% features. Furthermore, we additionally built an alternative model by excluding ADNP Spike to examine whether the separation between the groups would be achieved in the absence of this feature and to identify the strongest surrogate of ADNP Spike that can discriminate subjects between the 2 groups. These analyses were performed using R package “ropls” version 1.20.0 (69) and “glmnet” version 4.0.2 (70).

Correlations were performed using Spearman method followed by Benjamini-Hochberg multiple correction (71). The cocorrelate network was generated using R package “network” version 1.16.0 (72) and the chord diagram was generated using R package circlize version 0.4.10 (73).

A *P* value less than 0.05 was considered significant.

### Study approval

The studies were approved by the University of Washington Human Subjects IRB, and all participants, or their legally authorized representatives, completed informed consent.

## Author contributions

CS, KKQY, SF, CA, and GA wrote the manuscript with contributions from all authors. HYC, AW, RYC, and DMK enrolled the clinical cohorts and facilitated access to blood and plasma samples. CRW and JKL facilitated sample selection and analyzed the demographic and clinical data. CRW, JKL, KS, and NF facilitated subject enrollment, including collection and processing of the samples with assistance from EDL, MSA, KKQY, and CS. EDL, MSA, KKQY, and CS designed and executed the T cell experiments and analyzed the data. SF, CA, JY, and DHB performed the antibody experiments and analyzed the data. MTS analyzed T cell data and visualized T cell and antibody data. CL, DC, and DL facilitated computational analysis, including integrated analysis of T cell and antibody data. Co–first author order assignment was determined by the contribution amount of manuscript preparation, experimental design and execution, and data analysis.

## Supplementary Material

Supplemental data

Supplemental Table 1

## Figures and Tables

**Table 1:**
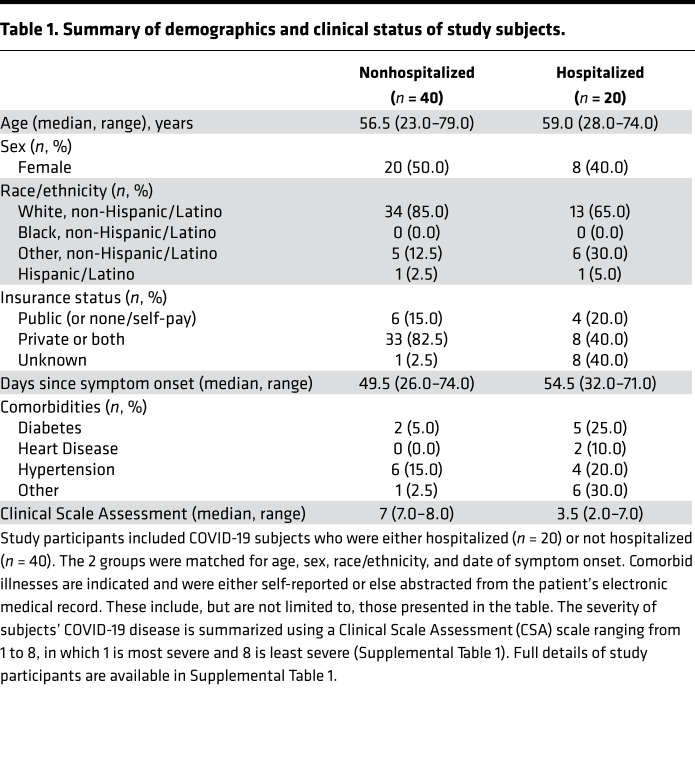
Summary of demographics and clinical status of study subjects.

## Version 1 02/23/2021

In-Press Preview

## Version 2 03/22/2021

Electronic publication
