# Supplementary material for: Comorbid illnesses are associated with altered adaptive immune responses to SARS-CoV-2
Source: JCI Insight. 2021 Mar 22;6(6):e146242. doi: 10.1172/jci.insight.146242 (PMC8026190; doi:10.1172/jci.insight.146242)
Supplement: Supplemental data [file jciinsight-6-146242-s086.pdf]

SUPPLEMENTAL METHODS

Sample processing

All whole blood patient samples were collected in acid citrate dextrose or sodium heparin tubes (one subject) and immediately transferred to the University of Washington. Whole blood was centrifuged at 200xg for 10 minutes to separate plasma. Plasma was collected, centrifuged at 1200xg to remove debris, aliquoted, and stored at -20ºC. Hank's balanced salt solution (HBSS) (Thermo Fisher Scientific, Waltham, MA) or 1x phosphate buffered saline (PBS) (Thermo Fisher Scientific, Waltham, MA) was added to the whole blood cellular fraction to replace plasma volume. Peripheral blood mononuclear cells (PBMC) were isolated by density-gradient centrifugation using Histopaque (Sigma-Aldrich, St. Louis, MO). After washing, purified PBMC were resuspended in 90% heat-inactivated fetal bovine serum (FBS) (Sigma-Aldrich, St. Louis, MO) with 10% dimethyl sulfoxide (DMSO) (Sigma-Aldrich, St. Louis, MO) cryopreservation media and stored in liquid nitrogen until use. Both plasma and PBMC were frozen within six hours of collection time.

Antibody neutralization

The SARS-CoV-2 pseudoviruses expressing a luciferase reporter gene were generated in an approach similar to as described previously (Tartof et al. 2020; Pujadas et al. 2020; Mathew et al. 2020). Briefly, the packaging plasmid psPAX2 (AIDS Resource and Reagent Program, Germantown, MD), luciferase reporter plasmid pLenti-CMV Puro-Luc (Addgene, Watertown, MA), and spike protein expressing pcDNA3.1-SARS CoV-2 SΔCT were co-transfected into HEK293T cells by lipofectamine 2,000 (Thermo Fisher Scientific, Waltham, MA). The supernatants containing the pseudotype viruses were collected 48 hours post-transfection, which were purified by centrifugation and filtration with a 0.45 µm filter. To determine the neutralization activity of the serum or plasma samples from cohorts, HEK293T-hACE2 cells were seeded in 96-well tissue

culture plates at a density of 1.75 x 10^4 cells/well overnight. Three-fold serial dilutions of heat inactivated (56°C for 30 minutes) serum or plasma samples were prepared and mixed with 50 µL of pseudovirus. The mixture was incubated at 37ºC for 1 hour before adding to HEK293T-hACE2 cells. 48 hours after infection, cells were lysed in Steady-Glo Luciferase Assay (Promega, Madison, WI) according to the manufacturer's instructions. SARS-CoV-2 neutralization titers were defined as the sample dilution at which a 50% reduction in relative light unit (RLU) was observed relative to the average of the virus control wells.

Antibody titer measurements and FcR binding

In order to measure antigen-specific antibody subclass, isotype, and Fc-receptor (FcR) binding levels, a customized multiplexed Luminex assay was utilized, as previously described (Brown et al. 2012). This allows for relative quantification of antigen-specific humoral responses in a high-throughput manner and detection of different antigens at once. A panel of SARS-CoV-2 antigens including the full spike glycoprotein (S) (provided by Eric Fischer, Dana-Farber Cancer Institute, Boston, MA), receptor binding domain (RBD) (Provided by Aaron Schmidt, Ragon Institute, Boston, MA) nucleocapsid (N) (Aalto Bio Reagents, Dublin, Ireland), S1 (Sino Biological, Beijing, China) and S2 (Sino Biological, Beijing, China) were used. In brief, antigens were coupled to uniquely fluorescent magnetic carboxyl-modified microspheres (Luminex Corporation, Austin, TX) using 1-Ethyl-3- (3-dimethylaminopropyl) carbodiimide (EDC) (Thermo Fisher Scientific, Waltham, MA) and Sulfo- N-hydroxysuccinimide (NHS) (Thermo Fisher Scientific, Waltham, MA). Antigen-coupled microspheres were then blocked, washed, and incubated for 16 hours at 4°C while rocking at 700 rpm with diluted plasma samples (1:1,000 for Fc-receptors, 1:500 for IgG1, and 1:100 for all other readouts) to facilitate immune complex formation. The following day, plates were washed using an automated plate washer (Tecan, Männedorf, Zürich, Switzerland) with 0.1% BSA and 0.02% Tween-20. Antigen-specific antibody titers were detected with Phycoerythrin (PE)-coupled antibodies against IgG1, IgG2, IgG3, IgG4, IgA, and IgM

(SouthernBiotech, Birmingham, AL). To measure antigen-specific Fc-receptor binding, biotinylated Fc-receptors (FcR2AH, 2B, 3AV, and 3B, Duke Protein Production facility) were coupled to PE and then added to immune-complexed beads to incubate for 1 hour at room temperature while shaking. Fluorescence was detected using an Intellicyt iQue with a PAA robot arm and analyzed using Forecyt software. The readout was mean fluorescence intensity (MFI) of PE. All experiments were performed in duplicate while operators were blinded to study group assignment, and all cases and controls were run at the same time to avoid batch effects.

Flow cytometry of T cells – surface marker staining

PBMC were plated at a density of up to 4×10^6 cells/well in a 96-well U-bottom plate and washed twice with PBS (Gibco, Waltham, MA). The cells were then stained with Fixable Green Live/Dead (Life Technologies, Carlsbad, CA) according to manufacturer's instructions and incubated for 15 minutes at room temperature. Live/Dead staining and all following steps were performed in the dark. At the end of the incubation, cells were washed twice in PBS and blocked by incubating the cells at 4°C for 15 minutes in a 1:1 mixture of human serum (Valley Biomedical, Winchester, VA) and FACS buffer (PBS supplemented with 0.2% bovine serum albumin (BSA) (Sigma, St. Louis, MO) sterile-filtered). Cells were stained with anti-CCR7 (clone 150503) (BD Biosciences, San Jose, CA) in the presence of 50 nM dasatinib (Cayman Chemicals, Ann Arbor, MI) at 37°C for 30 minutes. At the end of the incubation, the cells were washed and resuspended in FACS buffer containing 50 nM dasatinib with MR1-5-(2-oxopropylideneamino)- 6-D-ribitylaminouracil (5-OP-RU) and CD1d-α-Galactosylceramide (α-GalCer) tetramers (National Institutes of Health Tetramer Core Facility, Atlanta, GA) for 60 minutes at room temperature. Following the tetramer stain, the cells were washed twice in FACS buffer and stained at 4°C for 30 minutes with an antibody cocktail prepared in FACS buffer supplemented with 1 mM ascorbic acid and 0.05% sodium azide (Le Roy et al. 2009). Antibodies included anti-CD3 ECD (clone UCHT1) (Beckman Coulter, Brea, CA), anti-CD4 APC-H7 (clone L200), anti-CD8α BB700 (clone

RPA-T8), anti-CD38 BV605 (clone HB7), anti-CD45RA BUV737 (clone HI100), anti-HLA-DR BUV395 (clone G46-6) (BD Biosciences, San Jose, CA), anti-CD14 BV650 (clone M5E2), anti-CD19 BV785 (clone SJ25C1), anti-CD56 PE-Cy5 (clone HCD56), anti-TCR Vδ2 Alexa Fluor 700 (clone B6) (BioLegend, San Diego, CA), and anti-TCR γ/δ PE-Vio770 (clone 11F2) (Miltenyi Biotech, Auburn, CA). The samples were subsequently washed with FACS buffer and PBS. Cells were then fixed in 1% paraformaldehyde (Electron Microscopy Sciences, Hatfield, PA) and PBS solution for 15 minutes at 4°C, washed, and resuspended in PBS containing 2 mM ethylenediaminetetraacetic acid (EDTA) and stored at 4°C until acquisition. Samples were acquired on a BD LSRFortessa (BD Biosciences, San Jose, CA) equipped with a high-throughput sampler and configured with blue (488 nm), green (532 nm), red (628 nm), violet (405 nm), and ultraviolet (355 nm) lasers using standardized good clinical laboratory practice procedures to minimize variability of data generated.

SUPPLEMENTAL DATA

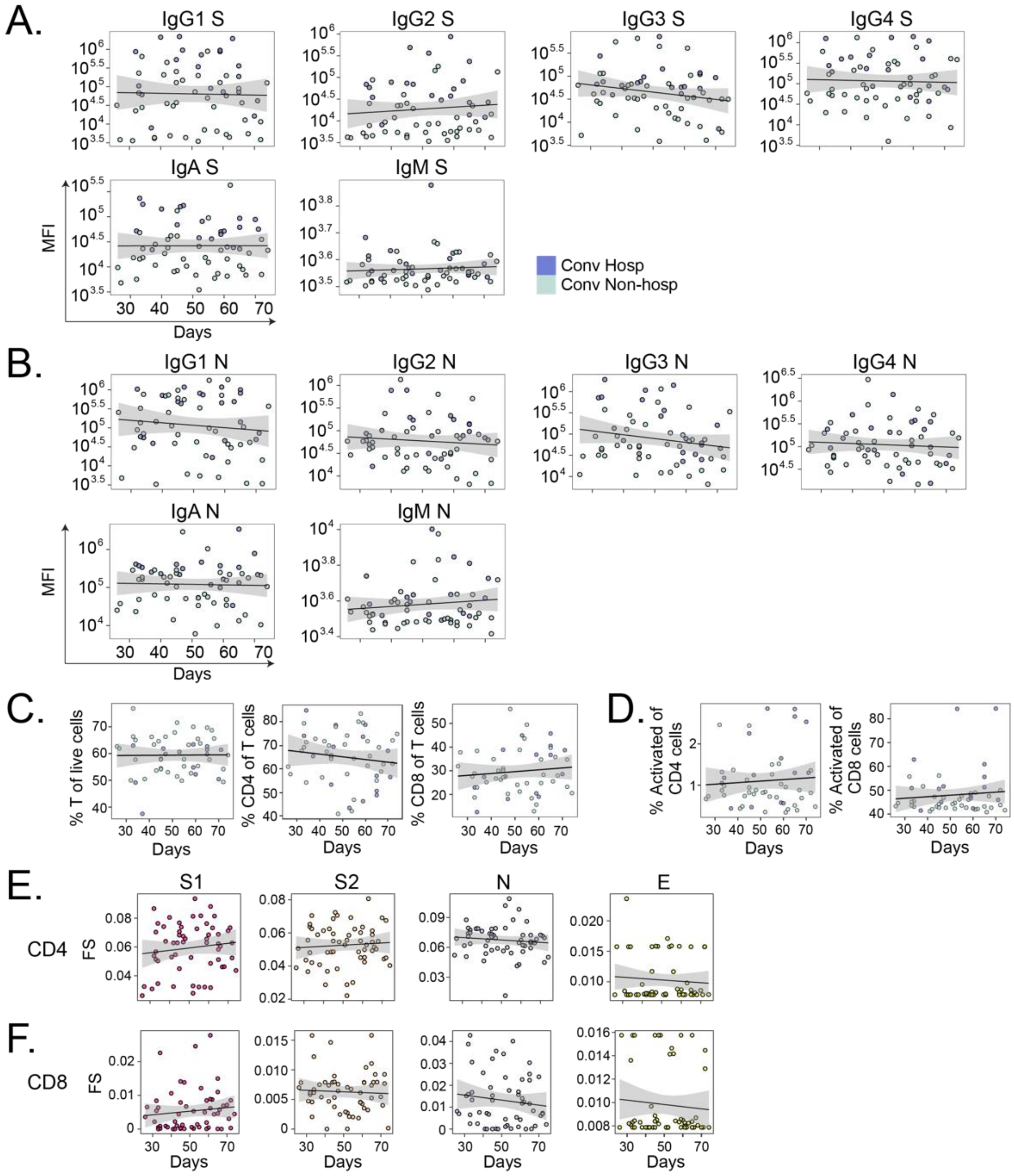

Supplemental Figure 1. Stability of SARS-CoV-2 specific antibody subclass and T cell frequencies over time. Magnitudes of (A) spike (S) and (B) nucleocapsid (N) specific antibodies are plotted in hospitalized (purple) and non-hospitalized (green) subjects by days since symptom onset and stratified by immunoglobulin subclass (IgM, IgG1, IgG2, IgG3, IgG4, and IgA). (C) Flow cytometric analysis comparing the percent of total CD3+ T cells graphed according to days since symptom onset. Among CD3+ T cells, the percent of CD4+ and CD8+ T cells is shown according to days since symptom onset. (D) The frequency of activated CD4+ and CD8+ T cells defined by co-expression of HLA-DR and CD38 are graphed according to date of symptom onset. (E) The CD4 and (F) CD8 T cell functionality scores (FS) were determined by COMPASS. For each stimulation, we examined the association of days since symptom onset. The black lines on the scatter plots represent best fit linear regression lines, and the grey-shaded areas represent the 95% confidence interval of the predicted means. All p-values are not significant, indicating that the measured responses do not change over time. n = 60 in all panels.

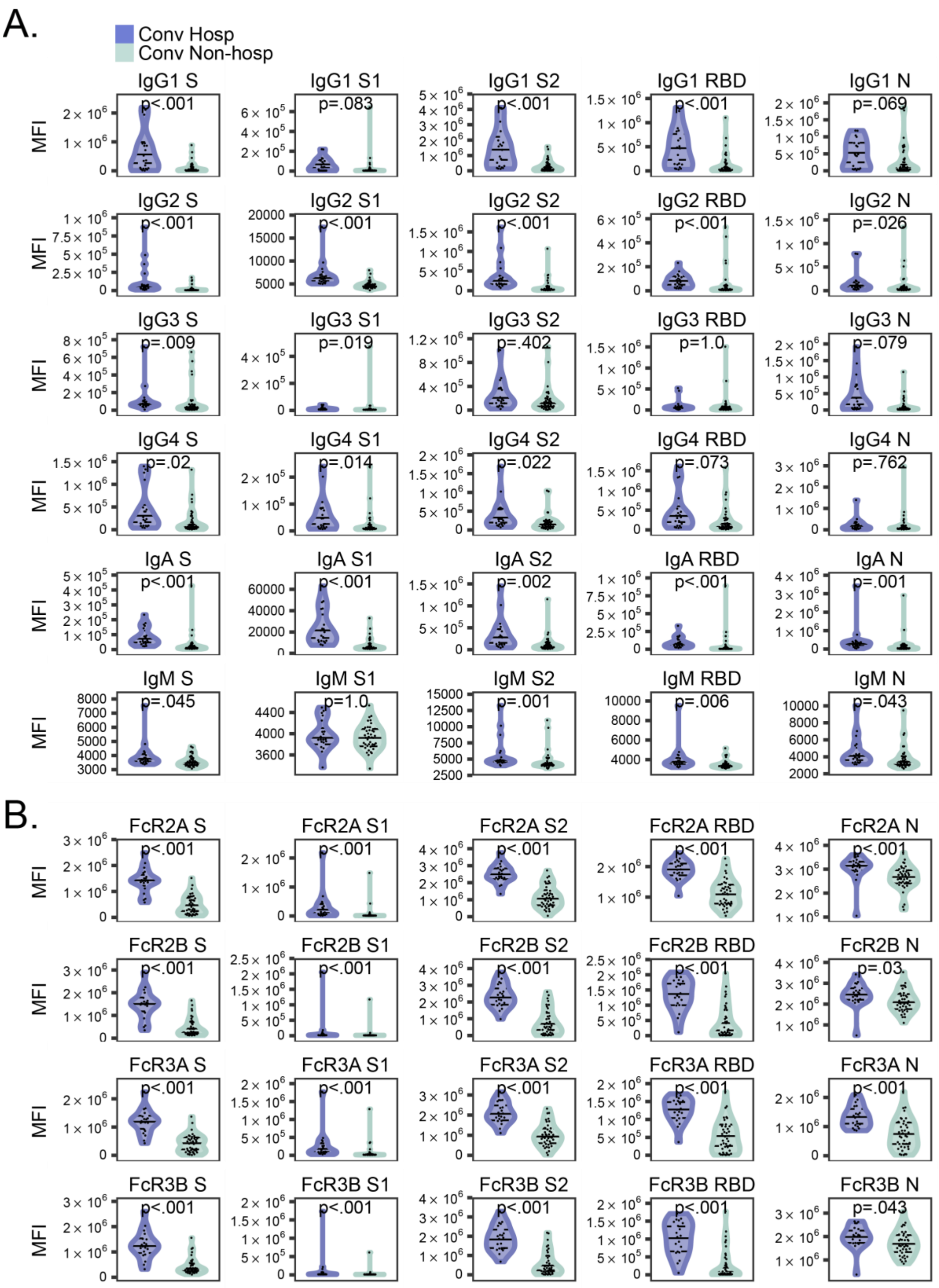

Supplemental Figure 2. Univariate analysis of antigen-specific antibody responses. Violin plots show all features measured via the antigen-specific customized luminex assay. The readout is mean fluorescence intensity (MFI), indicating relative antibody titer. In each graph, MFI was compared between hospitalized (purple, n=20) and non-hospitalized (green, n=40) subjects using a Mann-Whitney test and corrected for multiple hypothesis testing using the Bonferroni method. In total, 50 variables were measured: (A) IgG1, IgG2, IgG3, IgG4, IgA, and IgM, and (B) FcR2A, FcR2B, FcR3A, and FcR3B against spike (S), receptor binding domain (RBD), and nucleocapsid (N) antigens.

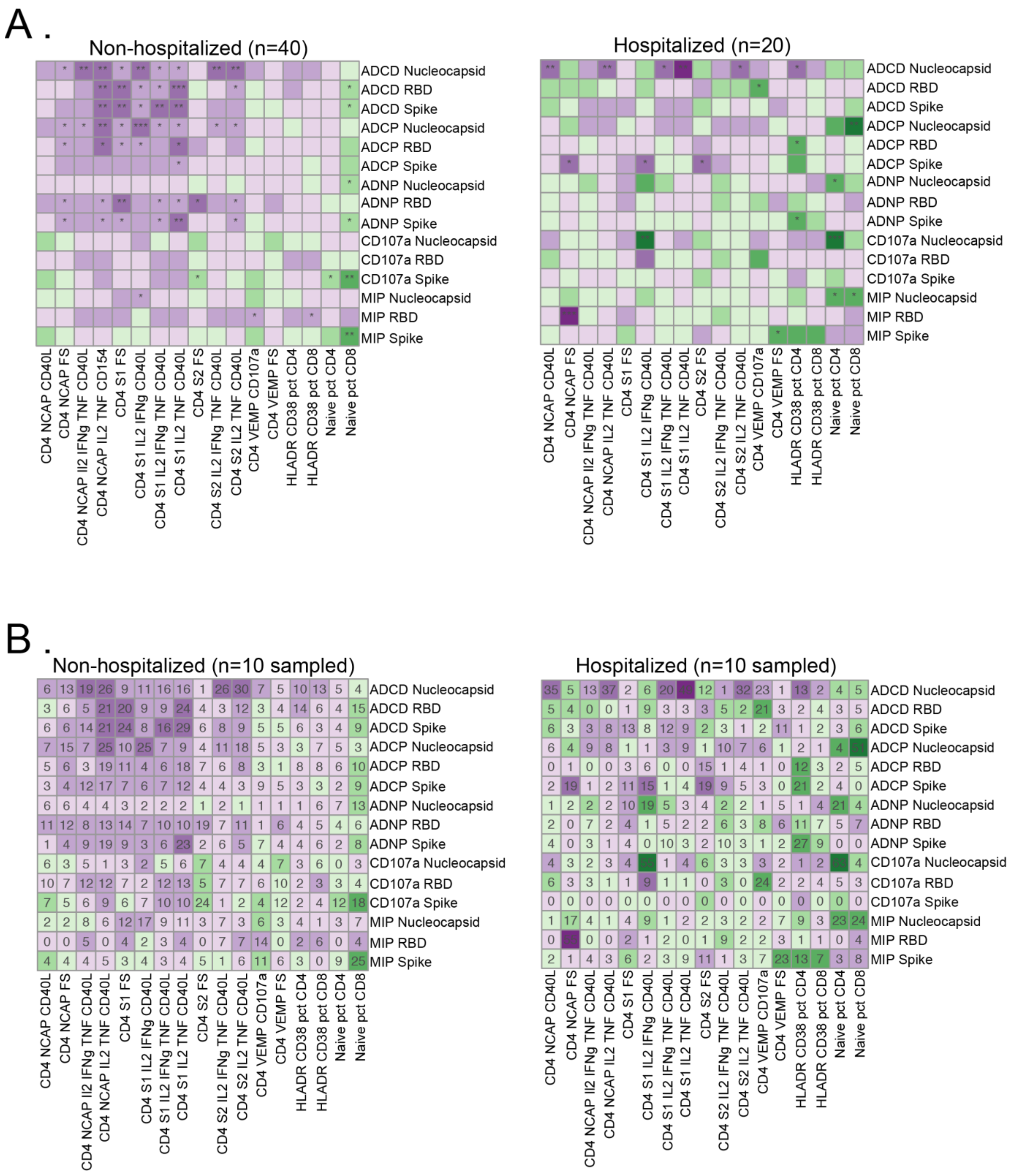

Supplemental Figure 3. Correlations between the antibody and T-cell features are robust to sample size. (A). Heatmaps show Spearman correlations of antibody functions (in rows) with T-cell responses (in columns) using 40 non-hospitalized and 20 hospitalized subjects. The color of each cell is associated with the correlation coefficient, and the significance of correlation is denoted with asterisks (*p<0.5, **p<0.01, ***p<0.001). (B) To exclude potential bias caused by the number of subjects, 10 subjects were sampled per group. This was repeated 100 times, and the average of the Spearman correlation coefficient was taken for each functional antibody feature-T cell measurement pair. The color of each cell is associated with the average correlation coefficient, and the numbers in the cell denote the number of times the correlation was significant (p < 0.05).

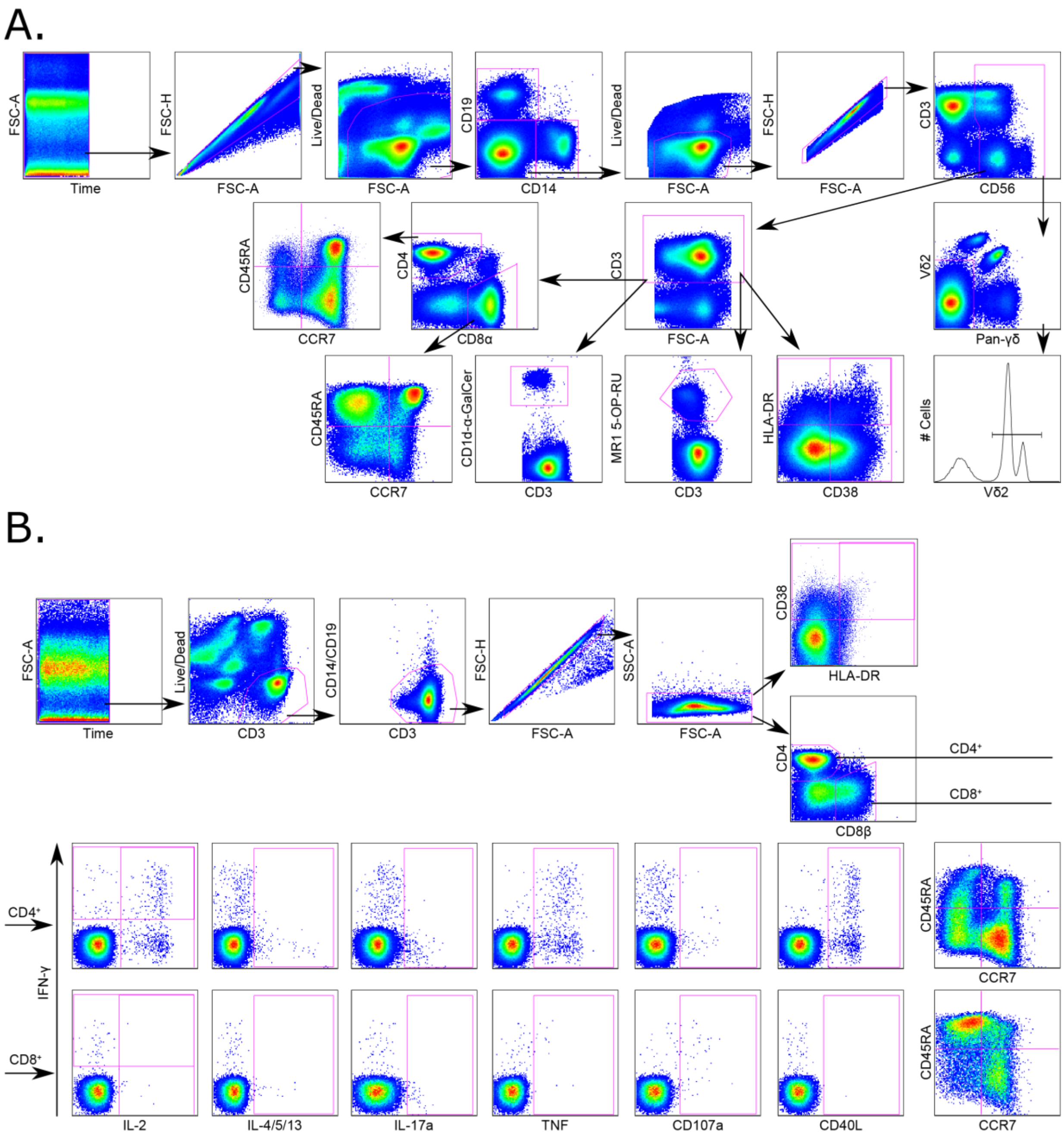

Supplemental Figure 4. Gating strategy for T cell flow cytometry. (A) Data presented in Figure 1 were obtained using a 15-color multiparameter flow cytometry panel. Events were first isolated from a time gate, followed by singlets. Viable cells were identified, and then CD19 and CD14 markers were used to identify B cells and monocytes, respectively. Gating then proceeded from lymphocytes to a second singlet gate. CD56 was then used to identify natural killer cells. In parallel, CD3+ T cells from the singlet gate were further characterized using CD1d-α-Galactosylceramide (α-GalCer) and MR1-5-(2-oxopropyl phenylamino)-6-D-ribitylaminouracil (5-OP-RU) tetramers to identify invariant natural killer T cells and mucosal-associated invariant T cells, respectively, as well as activation markers (HLA-DR and CD38), and γδ T cells (Pan-γδ and Vδ2). In addition, CD3+ T cells were also examined for co-receptor usage with CD4 and CD8 markers. Finally, memory populations were separately gated for CD4+ and CD8+ cells using CD45RA and CCR7. (B) Data presented in Figures 3-5 were obtained using a 14-color multiparameter intracellular cytokine staining flow cytometry panel. A time gate was applied to the events, and then viable CD3+ T cells were identified. CD14 and CD19 markers were used to exclude monocytes and B cells, and then a singlet gate was applied. Lymphocytes were gated and analyzed for HLA-DR (activation), CD38 (activation), and CD4 and CD8 co-receptor expression. For CD4+ and CD8+ populations, cells were characterized for expression of IFN-γ (Th1), IL-2 (Th1), TNF (Th1), IL4/5/13 (Th2), IL-17 (Th17), CD40L (activation and B cell help), CD107a (degranulation), CD45RA (memory), and CCR7 (memory) expression.

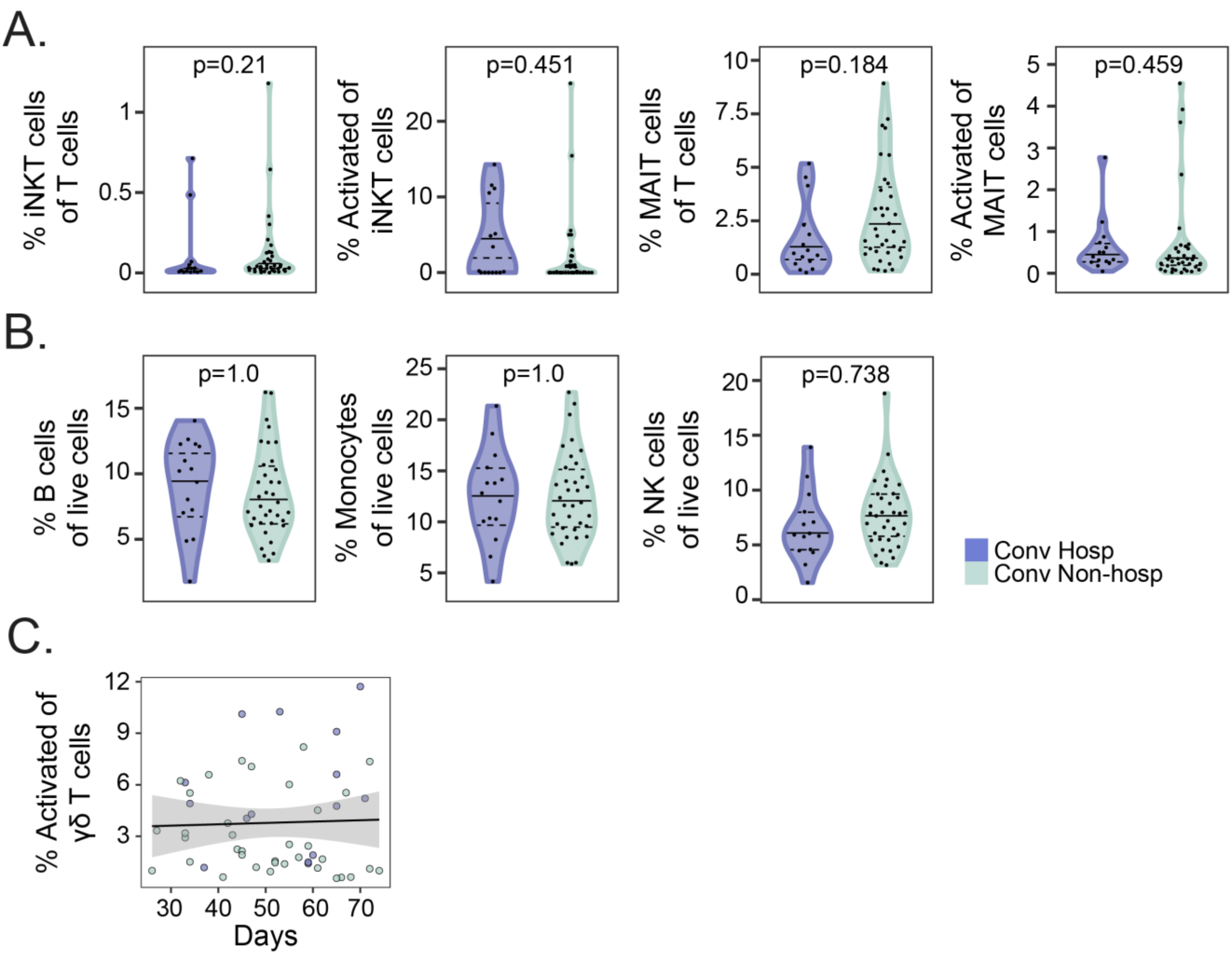

Supplemental Figure 5. Cell frequencies of donor-unrestricted T cells, B cells, monocytes, and natural killer cells. Flow cytometric analysis of peripheral blood mononuclear cells (PBMC) was performed using a 15-color surface staining and phenotyping panel. (A) Frequencies and activation statuses of invariant natural killer T (iNKT) cells and mucosal-associated invariant T (MAIT) cells were compared between hospitalized (purple) and non-hospitalized (green) subjects. Frequencies are displayed as percent of total T cells, and activation is calculated as the percent total iNKT or MAIT cells that co-expressed HLA-DR and CD38. (B) B cells (CD19+), monocytes (CD14+), and natural killer (NK) cell (CD3-CD56+) frequencies are shown as percent of live cells and are compared between groups. (C) The frequency of activated (HLADR+CD38+) γδ T cells is plotted against days since symptom onset for both hospitalized and non-hospitalized subjects. T cell frequencies were compared between groups using Mann-Whitney U tests, followed by correction for multiple hypothesis testing using the Bonferroni method. Median, 25th, and 75th quartiles are indicated in the violin plots. The black line on the scatter plot represents a best fit linear regression line, and the grey-shaded area represents the 95% confidence interval of the predicted mean. If not shown, p-values were not significantly different. n = 60 in all panels.

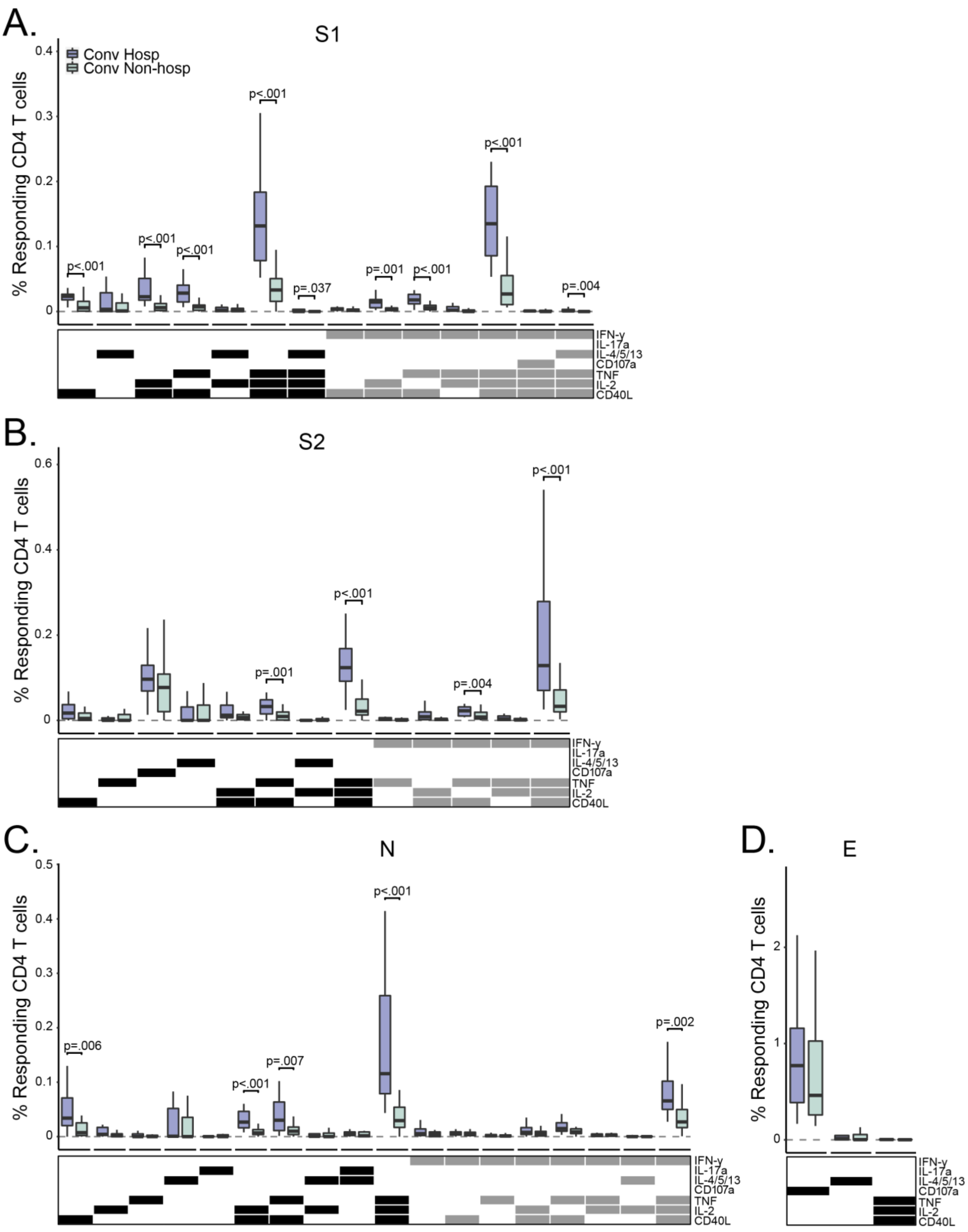

Supplemental Figure 6. Convalescent COVID-19 subjects demonstrate both IFN-γ dependent and independent CD4+ T cell responses following stimulation with SARS-CoV-2 protein antigens. Background subtracted magnitudes of responding CD4 T cells is displayed for each of the functional subsets identified by COMPASS in Figure 3A after stimulation with peptide pools targeting (A) S1, (B) S2, (C) nucleocapsid, and (D) envelope. Boxplots indicating median and interquartile range are shown for hospitalized (purple, n=20) and non-hospitalized (green, n=40) subjects. Cell frequencies were compared between groups using Mann-Whitney U tests, followed by correction for multiple hypothesis testing using the Bonferroni method. Only significant p-values are indicated.

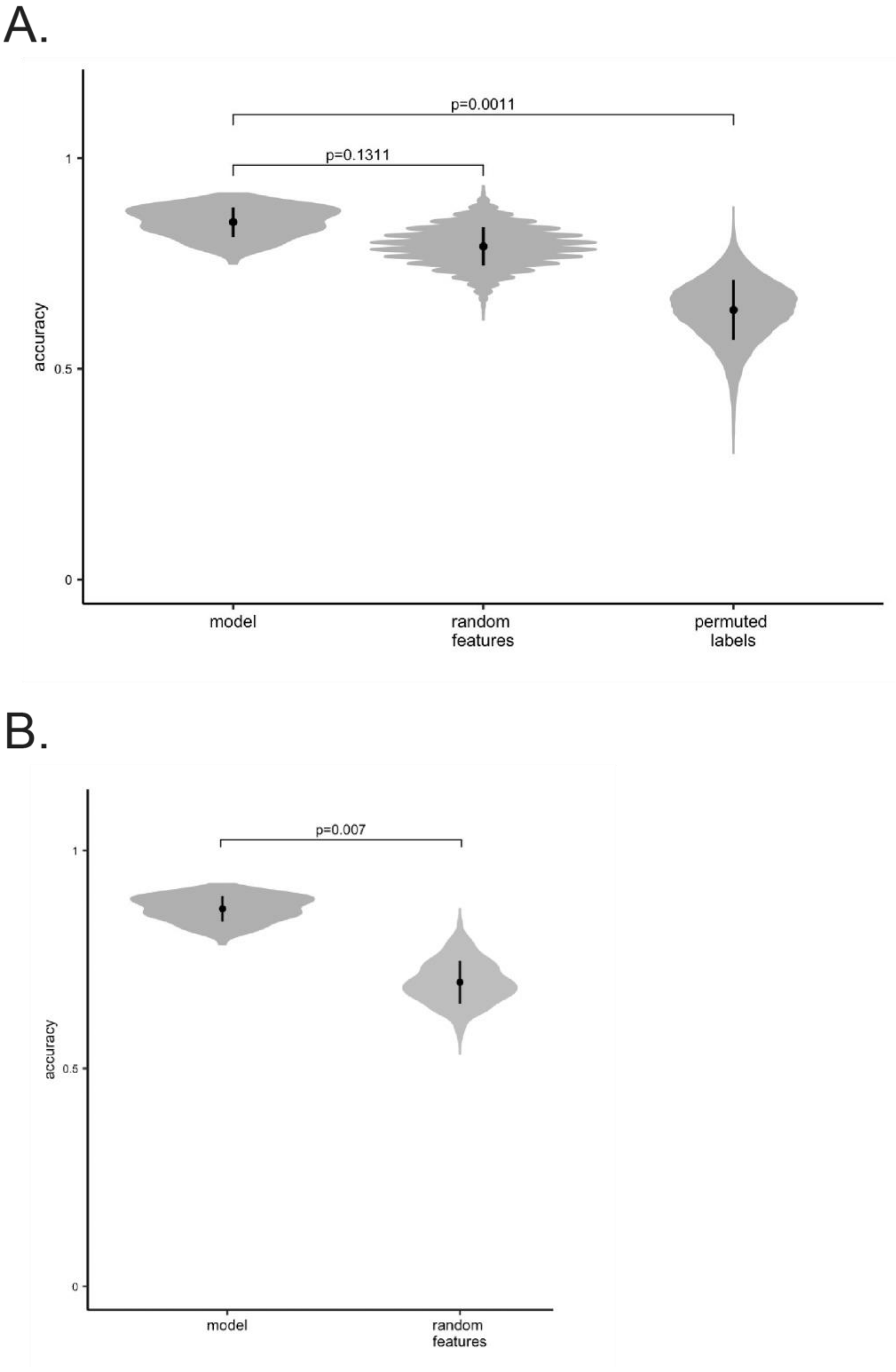

Supplemental Figure 7. Validation of PLS-DA model. The classification accuracy distributions of the model presented in Figure 6 were compared to negative control models based on randomly selected or permuted data by measuring the classification accuracies of each model in a five-fold cross-validation framework (n=60). (A) The violin plot shows the distributions of these classification accuracies for all three models across cross-validation replicates. Model performs significantly better compared to permuted labels. The model is not able to outperform the randomly selected features because a substantial portion of the measured features (54%) are significantly correlated (Spearman correlations, Benjamini-Hochberg adjusted p-value < 0.05) with a LASSO-selected feature, ADNP Spike, thus are replaceable with ADNP Spike. (B) Features that are correlated with ADNP Spike were excluded. The model performs significantly better compared to randomly selected features from the pool of features, which are not significantly correlated with ADNP spike.
